# SIRT3 mediates CPT2 delactylation to enhance mitochondrial function and proliferation in goat granulosa cells

**DOI:** 10.1186/s40104-025-01231-8

**Published:** 2025-07-17

**Authors:** Shuaifei Song, Mingzhi Yang, Jiayue Li, Yaru Li, Lei Wang, Shiyi Yao, Zihan Wang, Qiuyan Li, Yanguo Han, Dejun Xu, Zhongquan Zhao

**Affiliations:** Chongqing Key Laboratory of Herbivore Science, College of Animal Science and Technology, Southwest University, Chongqing, 400715 China

**Keywords:** CPT2, Delactylation, Mitochondrial function, Ovarian granulosa cells, Proliferation, SIRT3

## Abstract

**Background:**

Reproductive efficiency in goats is closely linked to the healthy development of follicles, with the proliferation of ovarian granulosa cells (GCs) playing a crucial role in this process. Sirtuin 3 (SIRT3), an enzyme that catalyzes post**-**translational modifications (PTMs) of proteins, is known to regulate a variety of mitochondrial metabolic pathways, thereby affecting cell fate. However, the specific effect of SIRT3 on the follicular development process remains unclear. Therefore, this study aimed to investigate the regulatory role of SIRT3 in the mitochondrial function and proliferation of goat GCs, as well as the underlying mechanisms involved.

**Results:**

In this study, GCs from small follicles in goat ovaries presented increased proliferative potential and elevated SIRT3 expression levels compared with those from large follicles. In vitro, SIRT3 overexpression enhanced mitochondrial function, promoted proliferation and inhibited apoptosis in GCs. Correspondingly, the inhibition of SIRT3 led to the opposite effects. Notably, SIRT3 interacted with carnitine palmitoyl transferase 2 (CPT2) and stabilized the CPT2 protein by mediating delactylation, which prolonged the half**-**life of CPT2 and prevented its degradation. Further investigation revealed that CPT2 overexpression enhanced fatty acid β**-**oxidation and mitochondrial function in GCs. Additionally, CPT2 promoted the proliferation of GCs by increasing the protein levels of β**-**catenin and its downstream target, cyclin D1 (CCND1). However, this effect was reversed by 3**-**TYP (a SIRT3 inhibitor).

**Conclusions:**

SIRT3 stabilizes CPT2 protein expression through delactylation, thereby enhancing mitochondrial function and the proliferative capacity of GCs in goats. This study provides novel insights into the molecular mechanisms and regulatory pathways involved in mammalian follicular development.

**Graphical Abstract:**

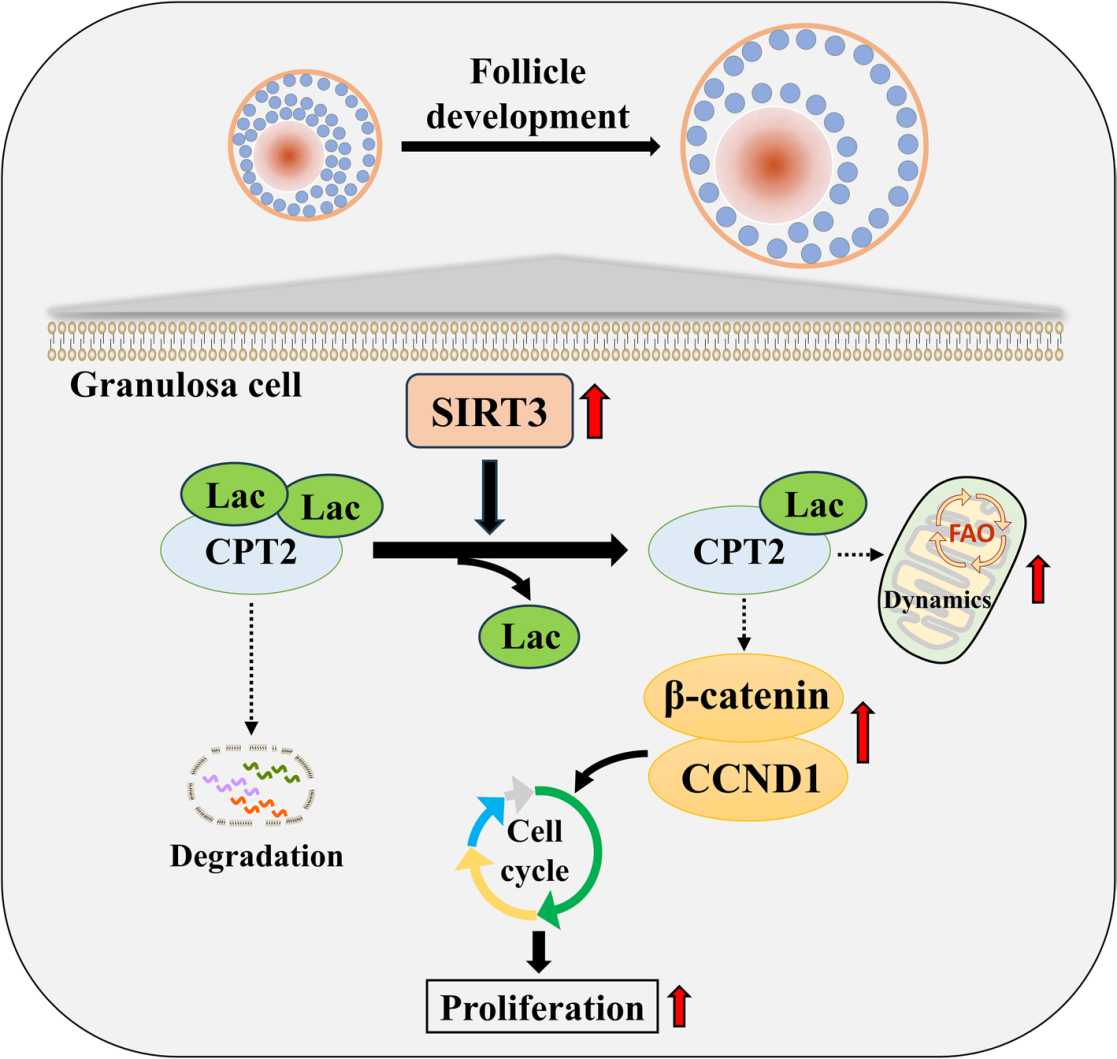

**Supplementary Information:**

The online version contains supplementary material available at 10.1186/s40104-025-01231-8.

## Introduction

Reproductive efficiency is a key determinant of livestock industry development, particularly in species with lower fertility, such as goats. The number of lambs per litter is an important trait that directly influences both the scale and economic benefits of goat farming [1]. The reproductive potential of mammals is closely linked to the number of mature oocytes that the ovaries can provide [2, 3]. As the fundamental functional unit of the ovary, the healthy development of follicles is essential for ensuring the release of high**-**quality oocytes. Goats typically ovulate no more than three times per oestrous cycle, which is the major limiting factor for their lambing rate [4]. Consequently, increasing the number of ovulations per cycle may be an effective strategy for improving goat fertility. However, the process by which follicles progress from the primordial stage to the mature stage is regulated by various complex factors [5]. A deeper understanding of these regulatory mechanisms can provide theoretical support for advancing goat reproductive performance.

Ovarian granulosa cells (GCs) are the primary components that form the follicular structure [6]. During follicular development, the hormones and proteins secreted by GCs are essential for oocyte growth and maturation [7]. Additionally, GCs can also provide energy and support to oocytes via gap junctions [2, 8]. Following the activation of primordial follicles, the rapid proliferation of GCs is required for subsequent follicular development and ovulation [9]. In contrast, loss of proliferative capacity in GCs during the early follicular stages accelerates follicular depletion and fertility decline in female mice [10]. An adequate ATP supply is considered a fundamental requirement for inducing GC proliferation, with mitochondria serving as the primary site of ATP production [11]. Recent studies have emphasized the importance of good mitochondrial function in supporting GC proliferation in mouse and hen follicles [12, 13]. Mitochondrial morphology and quality are regulated primarily by the dynamic processes of mitochondrial fission and fusion, which are known collectively as mitochondrial dynamics. Disruption of mitochondrial dynamics impairs GC proliferation, induces apoptosis, and leads to follicular atresia, ultimately reducing reproductive capacity [14].

Sirtuin 3 (SIRT3), a member of the Sirtuins family, is an NAD^+^-dependent deacetylase, and is predominantly localized in the mitochondrial matrix [15]. SIRT3 is a key regulator of mitochondrial energy metabolism and cellular fate [16]. Studies have demonstrated that SIRT3 expression in mouse ovaries decreases with age [17], which leads to severe mitochondrial dysfunction, metabolic disturbances, and cell apoptosis [18–20]. Conversely, the upregulation of SIRT3 in bovine GCs can improve mitochondrial function and mitigate damage [21]. However, there is still no direct evidence that SIRT3 regulates the function of goat ovarian granulosa cells. Interestingly, the functional effects of SIRT3 are mediated primarily through the deacetylation of its downstream target proteins. Additionally, SIRT3 has been identified as an "eraser" of lysine lactylation (K**-**Lac), which is a novel post**-**translational modification (PTM) [22]. It is known that SIRT3 may play important regulatory roles in mitochondrial function and GC proliferation during follicular development. However, it remains unclear whether SIRT3 influences the goat GC functions through regulating K**-**Lac and its potential downstream targets. Therefore, the objective of this study was to investigate the potential mechanisms by which SIRT3 regulates GC proliferation, elucidating its pivotal role in follicular development, thereby providing essential data for identifying candidate genes involved in goat reproduction and informing strategies for conservation breeding.

## Materials and methods

### Ethics statement

All animal procedures used in this study were approved by the Animal Ethics Committee of Southwest University, and the approval number is IACUC**-**20220915**-**01.

### Primary cell culture

Goat ovarian GCs were isolated based on a previously established method [23]. In brief, ovaries (*n* = 20) from slaughtered Dazu black goats were collected and washed sequentially with pre**-**warmed 0.9% NaCl solution at 37 °C, followed by 75% alcohol and phosphate**-**buffered saline (PBS, G4202, Servicebio, Wuhan, China). Follicular fluid from healthy follicles (1–6 mm in diameter) on the ovarian surface was subsequently collected and filtered through a cell strainer (70 µm, BS**-**70**-**CS, Biosharp, Hefei, China) into a 15**-**mL centrifuge tube (601052, Nest, Wuxi, China). GCs were obtained by centrifugation at 1,500 r/min for 5 min. The GCs were resuspended in DMEM/F12 (11330500, Gibco, CA, USA), which included 10% fetal bovine serum (FBS, sh30406, HyClone, Utah, USA) and 1% penicillin**-**streptomycin (BL505A, Biosharp, Hefei, China). Finally, the GCs were seeded in cell culture flasks and placed in an incubator at 37 °C with 5% CO_2_. The medium was replaced with fresh medium after 24 h.

### Cell transfection

Goat ovarian GCs were pre**-**cultured in 6**-**well plates for 24 h to reach a confluence of 60%–80% before treatment. For overexpression plasmid transfection, *SIRT3* and *CPT2* overexpression plasmids, as well as a vector plasmid, were synthesized (Genechem, Shanghai, China). Transfection was carried out using Lipofectamine™ LTX reagent and PLUS™ reagent (15338, Thermo Fisher Scientific, Shanghai, China). A total of 2.5 µg of plasmid per well was transfected into GCs in a 6**-**well plate for 48 h according to the manufacturer’s instructions. For RNA interference, *SIRT3* small interfering RNA (siRNA), *CPT2* siRNA, and negative control siRNA (Sangon Biotech, Shanghai, China) were transfected into GCs using RNATransMate (E607402, Sangon Biotech, Shanghai, China) at a final concentration of 20 nmol/L for 8 h. Afterwards, the medium was replaced with DMEM/F12 containing 10% FBS, and the cells were cultured for 48 h. Samples were collected for subsequent assays. The sequences of the siRNAs used are shown in Table 1.

**Table 1:** Sequence of siRNA (goat)

Gene	Sense (5'**→**3')	Antisense (5'**→-**3')
si-NC	UUCUCCGAACGUGUCACGUTT	ACGUGACACGUUCGGAGAATT
si-*SIRT3*-1	GCCACGGUCAGAAGAAGUUTT	AACUUCUUCUGACCGUGGCTT
si-*SIRT3*-2	CCAAUGCUACUCACUACUUTT	AAGUAGUGAGUAGCAUUGGTT
si-*SIRT3*-3	CCCUGACUCAAAGCUCGUUTT	AACGAGCUUUGAGUCAGGGTT
si-*CPT2*-1	GCUCAGGACAAGCAGAAUATT	UAUUCUGCUUGUCCUGAGCTT
si-*CPT2*-2	CUGUUGUCCUGAACUUUAATT	UUAAAGUUCAGGACAACAGTT
si-*CPT2*-3	GGUUUGACAAAUCCUUUAATT	UUAAAGGAUUUGUCAAACCTT

### Immunofluorescence assay

Fresh goat ovarian samples were fixed in 4% paraformaldehyde (P0099, Beyotime, Shanghai, China) overnight at 4 °C. The fixed tissues were subsequently embedded in paraffin and sectioned. After deparaffinization and antigen retrieval, the sections were blocked with 5% bovine serum albumin (BSA) for 1 h. The sections were then incubated with the target primary antibody (SIRT3, 1:300; CPT2, 1:300) overnight at 4 °C. The following day, the sections were incubated with a fluorescent secondary antibody (1:500) for 2 h at room temperature. For nuclear counterstaining, the sections were incubated with DAPI (C1005, Beyotime, Shanghai, China) in the dark for 15 min. Finally, the sections were sealed with an anti**-**fluorescence quenching agent (P0126, Beyotime, Shanghai, China). Images were captured using a fluorescence microscope (Zeiss, Axio Observer 3, Germany).

### Immunohistochemistry

Paraffin**-**embedded ovarian tissue sections were deparaffinized by sequential washes with xylene and graded alcohol. After antigen retrieval and treatment with 3% hydrogen peroxide, the sections were incubated with 5% normal goat serum for 1 h to block nonspecific binding. Next, the sections were incubated overnight at 4 °C with the target primary antibody and then incubated with the secondary antibody at room temperature. The sections were subsequently developed with DAB (P0202, Beyotime, Shanghai, China) and counterstained with haematoxylin. Finally, the slides were dehydrated, cleared, and mounted with coverslips. Immunostaining was evaluated using light microscopy.

### 5-Ethynyl-2'-deoxyuridine (EdU) assay

The proliferation ability of the GCs was evaluated using a BeyoClick™ EdU Cell Proliferation Kit with Alexa Fluor 594 (C0078, Beyotime, Shanghai, China). First, the GCs were seeded into 96**-**well plates at a density of 2 × 10^3^ cells per well, and EdU staining was performed following specific treatments. The procedure was performed according to the manufacturer’s instructions. Finally, the cells were visualized using a fluorescence microscope, and the percentage of EdU**-**positive cells was calculated.

### Cell cycle distribution assay

Treated cells in 6**-**well plates were collected by trypsin digestion (C0203, Beyotime, Shanghai, China) and fixed overnight at 4 °C in 70% ethanol. After being washed with PBS, the cells were incubated with propidium iodide (PI) staining solution at 37 °C for 30 min in the dark. The cell cycle distribution was analyzed by flow cytometry (BD Biosciences, CA, USA). The data were analyzed using ModFit software (Verity Software House, MA, USA).

### CCK-8 assay

A Cell Counting Kit**-**8 (CCK**-**8, C0038, Beyotime, Shanghai, China) was used to examine cell viability. GCs were seeded into 96**-**well plates at a density of 5 × 10^3^ cells per well. After treatment, 10 µL of CCK**-**8 solution was added to each well containing 100 µL of medium and incubated at 37 °C for 1 h. Finally, the optical density (OD) values were measured at a wavelength of 450 nm.

### Determination of the ROS level

The total reactive oxygen species (ROS) levels in GCs were detected using a ROS assay kit (S0033, Beyotime, Shanghai, China). Briefly, GCs were seeded into 24**-**well plates at a density of 1 × 10^4^ cells per well and treated as described. The cells were subsequently incubated with 10 µmol/L DCFH**-**DA dye at 37 °C for 20 min in the dark. After the GCs were washed with DMEM/F12, the level of ROS in the GCs was observed, and images were captured using a fluorescence microscope.

### Quantitative real-time PCR

Total RNA was extracted from GCs using RNAiso Plus (9109, TaKaRa, Kyoto, Japan), and the RNA concentration was measured using a NanoDrop spectrophotometer (Thermo Fisher Scientific, MA, USA). Total RNA (1 μg) was reverse transcribed using a PrimeScript™ FAST RT Reagent Kit with gDNA Eraser (RR092A, TaKaRa, Kyoto, Japan). Real**-**time PCR was performed with TB Green^®^ Premix Ex Taq™ II (RR820A, TaKaRa, Kyoto, Japan) on a CFX Connect Real**-**Time PCR Detection System (Bio**-**Rad, CA, USA). The program was as follows: predenaturation at 95 °C for 30 s, followed by 40 cycles of denaturation at 95 °C for 5 s and annealing at 60 °C for 30 s. The relative mRNA levels were normalized to those of *ACTB* and calculated using the 2^−ΔΔCt^ algorithm. All primers were designed using the National Center for Biotechnology Information (NCBI) database, and the sequences of the primers used in the experiments are listed in Table S1. Each sample was analyzed in triplicate.

### Western blot analysis

RIPA lysis buffer (P0013B, Beyotime, Shanghai, China) containing protease inhibitors (P1005, Beyotime, Shanghai, China) was used to lyse the treated GCs to extract total protein. In some experiments, the lysis buffer also contained a mixture of deacetylase inhibitors (P1112, Beyotime, Shanghai, China), and all steps were performed at 4 °C. The protein concentration was measured using a BCA protein assay kit (P0010, Beyotime, Shanghai, China). The protein samples were mixed with 5 × loading buffer (BL502, Biosharp, Hefei, China) and denatured by boiling at 100 °C for 5‒10 min. Protein separation was performed via 8%‒15% sodium dodecyl sulfate**–**polyacrylamide gel electrophoresis (SDS-PAGE), with 20 µg of protein loaded per lane. The separated proteins were then transferred to a polyvinylidene fluoride (PVDF) membrane (Millipore, MA, USA). After transfer, the membranes were blocked with 5% skim milk at room temperature for 2 h and incubated with specific primary antibodies at 4 °C overnight. After being washed with Tris**-**buffered saline containing 0.1% Tween**-**20 (TBST), the membranes were incubated with the corresponding secondary antibodies at room temperature for 2 h. The details of the antibodies used for the Western blot analysis are listed in Table S2. Finally, the protein bands were visualized on a gel imaging system (Bio**-**Rad, CA, USA) by the addition of an enhanced chemiluminescence (ECL) detection reagent (E411, Vazyme, Nanjing, China). Quantitative analysis of the bands was performed using Image J software.

### Immunoprecipitation and IP‒MS

Immunoprecipitation was performed using an immunoprecipitation kit with protein A + G magnetic beads (P2179, Beyotime, Shanghai, China) following the manufacturer’s instructions. Specifically, treated GCs were washed with PBS and lysed in lysis buffer containing a protease inhibitor. A total of 300 μL of the supernatant was used as a sample for immunoprecipitation, while the remaining supernatant was retained for the input group. SIRT3, CPT2, or IgG antibodies were each diluted to a working concentration of 20 µg/mL with TBS. Magnetic beads (10 μL suspension) were added to the antibody mixtures and incubated at room temperature for 2 h to allow the antibodies to bind to the beads. The antibody**-**bound beads were then incubated with the sample at 4 °C overnight. After washing three times with lysis buffer, the beads–protein complexes were resuspended in 1 × SDS-PAGE loading buffer and boiled at 95 °C for 5 min. Finally, the supernatants were collected for subsequent immunoblotting analysis.

For immunoprecipitation coupled with mass spectrometry (IP‒MS), as mentioned above, after SIRT3 overexpression, GCs were lysed, and protein samples were immunoprecipitated using magnetic beads conjugated with IgG or SIRT3 antibodies. The protein mixtures in the purified samples were analyzed by PTM BIO to identify interacting proteins. Candidate proteins that interact with SIRT3 in GCs were ultimately identified by excluding those detected in the IgG control group.

### Mitochondrial and cytosolic extraction

Cytoplasmic and mitochondrial proteins were extracted from GCs using a cell mitochondrial isolation kit (C3601, Beyotime, Shanghai, China) according to the manufacturer’s protocol. Briefly, treated GCs were collected, and 1 mL of mitochondrial separation reagent was added to 1 × 10^7^ cells. After a 15 min incubation on ice, the cell suspension was thoroughly homogenized using a glass homogenizer. After centrifugation at 600 × *g* for 10 min at 4 °C, the supernatant was collected and centrifuged again at 11,000 × *g* for 10 min at 4 °C. The supernatant from this second centrifugation was collected as the cytoplasmic fraction, while the mitochondrial pellet was resuspended in 100 μL of lysis buffer to isolate the mitochondrial proteins. These samples were stored and used for subsequent Western blot analysis. β**-**actin and VDAC1 were used as internal reference controls for cytoplasmic and mitochondrial proteins respectively, for Western blot analysis.

### Statistical analysis

All the data are presented as the mean ± SEM from at least three independent experiments unless otherwise specified. Statistical analyses were performed using GraphPad Prism 9 software (GraphPad Software, San Diego, CA, USA). Student’s *t*-test was used to compare the differences between two groups, and one**-**way analysis of variance was used for multiple comparisons. A *P* value < 0.05 was considered statistically significant.

## Results

### GCs derived from small follicles have high proliferation potential

**Fig. 1:**
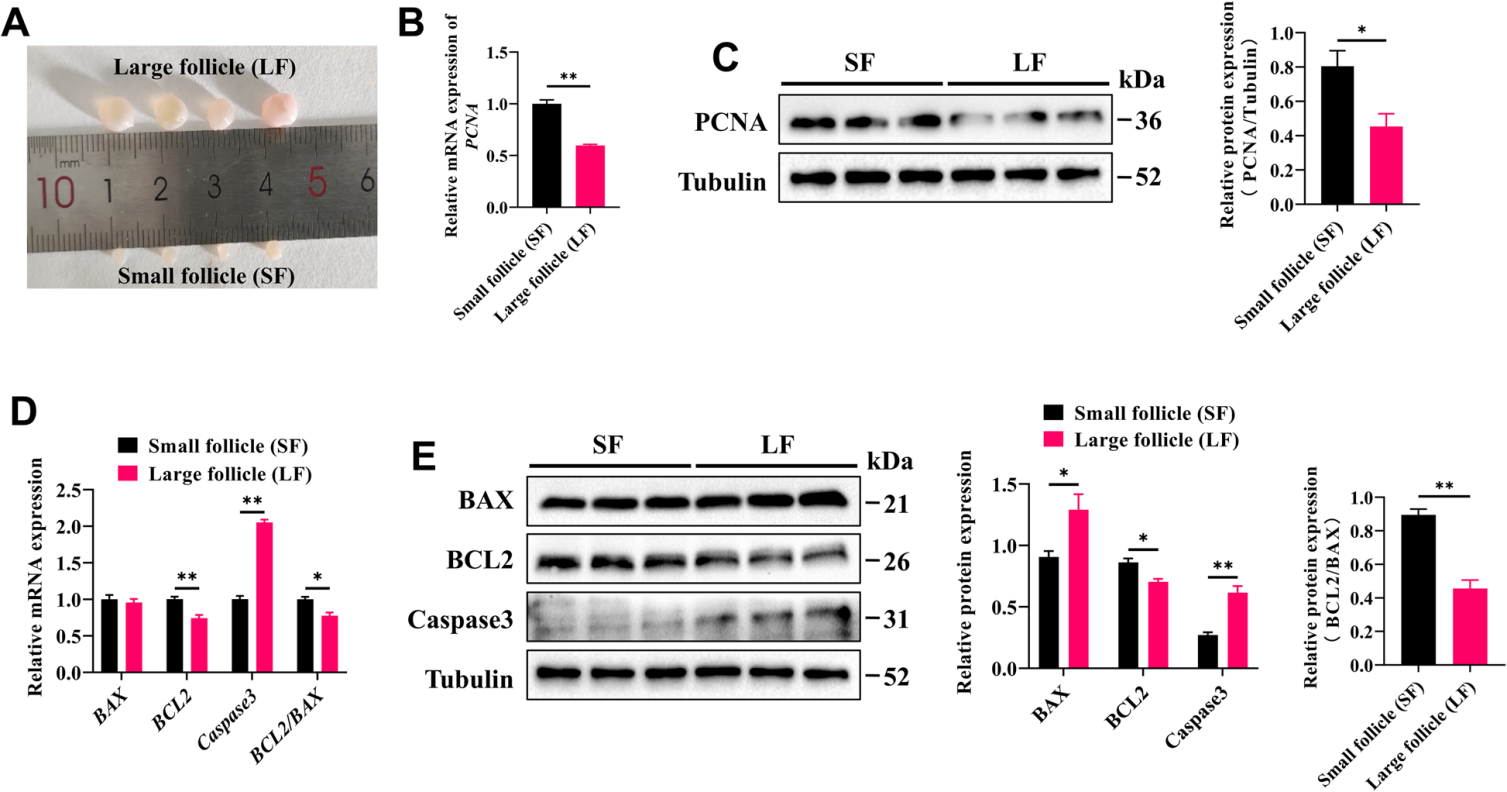
GCs derived from small follicles exhibited increased proliferation potential. **A** Representative image of small follicles (SF) and large follicles (LF) in goat ovaries. **B** The mRNA expression levels of *PCNA* in GCs from small and large follicles were quantified by RT-qPCR. **C** The protein expression level of PCNA in GCs was analyzed by Western blotting. **D** The mRNA expression levels of apoptosis**-**related genes, including *BAX*, *BCL2*, and *Caspase3*, were quantified by RT-qPCR. **E** Western blot analysis of apoptosis**-**related proteins in GCs. The data are presented as the mean ± SEM of three independent experiments. Statistical significance was determined using Student’s *t*-test, ^*^P < 0.05, ^**^P < 0.01

We classified the ovarian follicles of goats into small follicles (1–3 mm) and large follicles (> 3 mm) based on their diameter (Fig. 1A) [24]. To assess the proliferative potential of GCs from these follicles, GCs were isolated from both small and large follicles for subsequent analysis. Compared with those in GCs from small follicles, both the mRNA and protein levels of the cell proliferation marker PCNA were significantly downregulated in GCs derived from large follicles (Fig. 1B and C). Furthermore, the protein expression of the apoptosis**-**related factor BAX, as well as the mRNA and protein expression levels of Caspase3, were significantly increased in GCs from large follicles. In contrast, the mRNA and protein expression levels of BCL2, along with the BCL2/BAX ratio, were significantly reduced (Fig. 1D and E). These findings indicate that GCs from small follicles exhibit increased proliferative potential and a decreased apoptosis rate, which may contribute to the development of the follicle.

### SIRT3 is highly expressed in GCs from small follicles

**Fig. 2:**
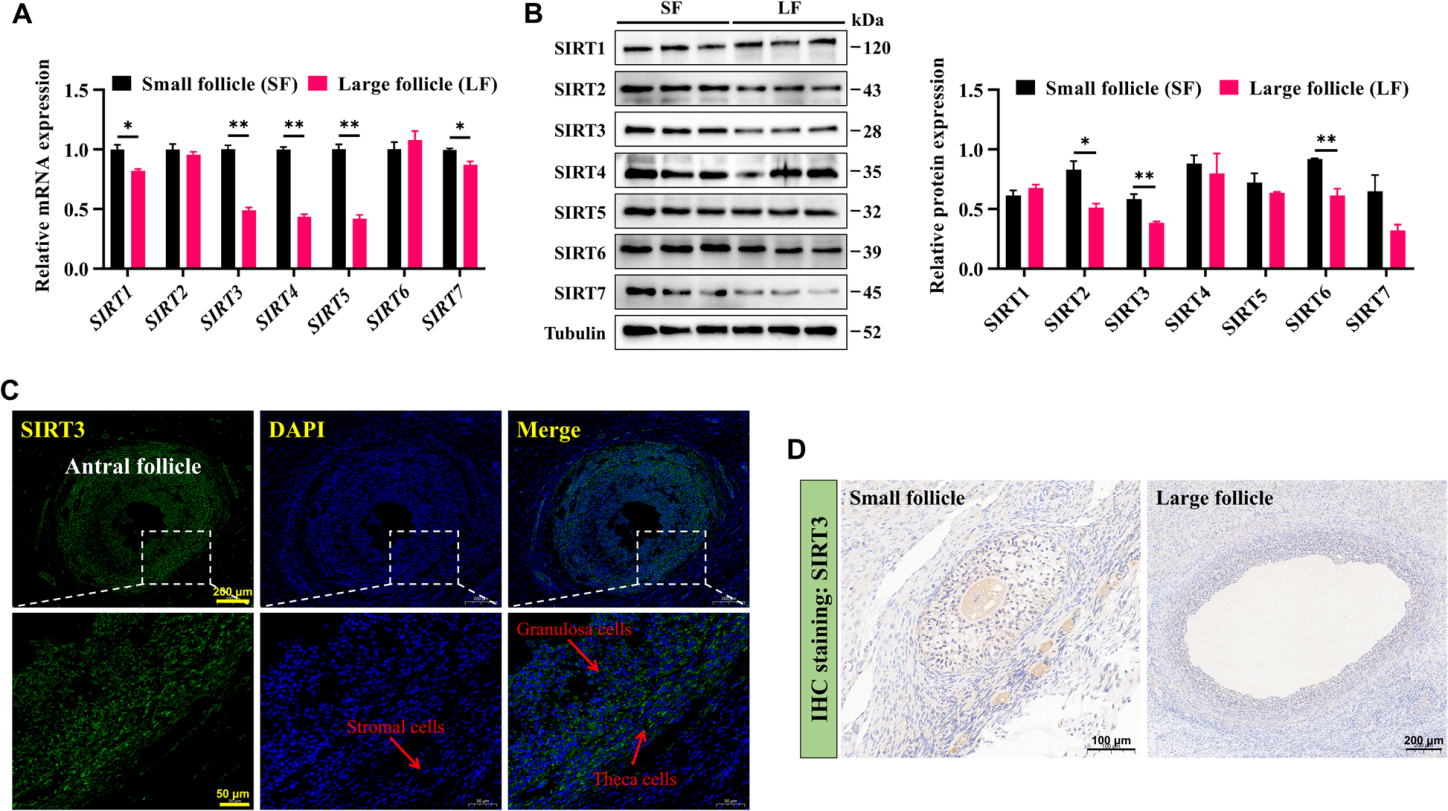
SIRT3 is highly expressed in GCs derived from small follicles. **A** The mRNA expression levels of Sirtuins (*SIRT1‒7*) in GCs from small and large follicles were quantified by RT-qPCR. **B** Relative protein expression levels of SIRT1‒7 in GCs from small follicles and large follicles. **C** Representative images of immunofluorescence staining for SIRT3 in goat ovaries; scale bar = 200 μm (top) and 50 μm (bottom). **D** Representative images of immunohistochemical staining for SIRT3 in goat ovaries; scale bar = 100 μm (left) and 200 μm (right). The data are presented as the mean ± SEM of three independent experiments. Statistical significance was determined using Student’s *t*-test, ^*^P < 0.05, ^**^P < 0.01

Sirtuins are involved in the regulation of various cellular functions, but their roles in follicular development remain unclear. To explore their potential involvement in this process, we assessed the expression of sirtuins in GCs from different follicle stages. RT-qPCR analysis revealed that, compared with those in large follicles, several sirtuins, including *SIRT1*, *SIRT3*, *SIRT4*, *SIRT5*, and *SIRT7*, exhibited higher mRNA levels in GCs from small follicles (Fig. 2A). Additionally, the protein levels of SIRT2, SIRT3, and SIRT6 were also elevated in small follicles (Fig. 2B). Notably, SIRT3 expression was significantly different at both the mRNA and protein levels, suggesting that SIRT3 may play a key regulatory role in follicular development. To further investigate this possibility, we performed immunofluorescence staining to determine the localization of SIRT3 in the goat ovary. The results revealed that SIRT3 was predominantly expressed in follicular cells, including GCs and theca cells (TCs) (Fig. 2C). Immunohistochemical staining further revealed that the positive staining (brown) intensity of SIRT3 in small follicular cells was greater (Fig. 2D), which echoed previous results. These findings suggest that SIRT3 may contribute to follicular development by regulating the function of GCs.

### SIRT3 promotes GC proliferation and inhibits apoptosis

**Fig. 3:**
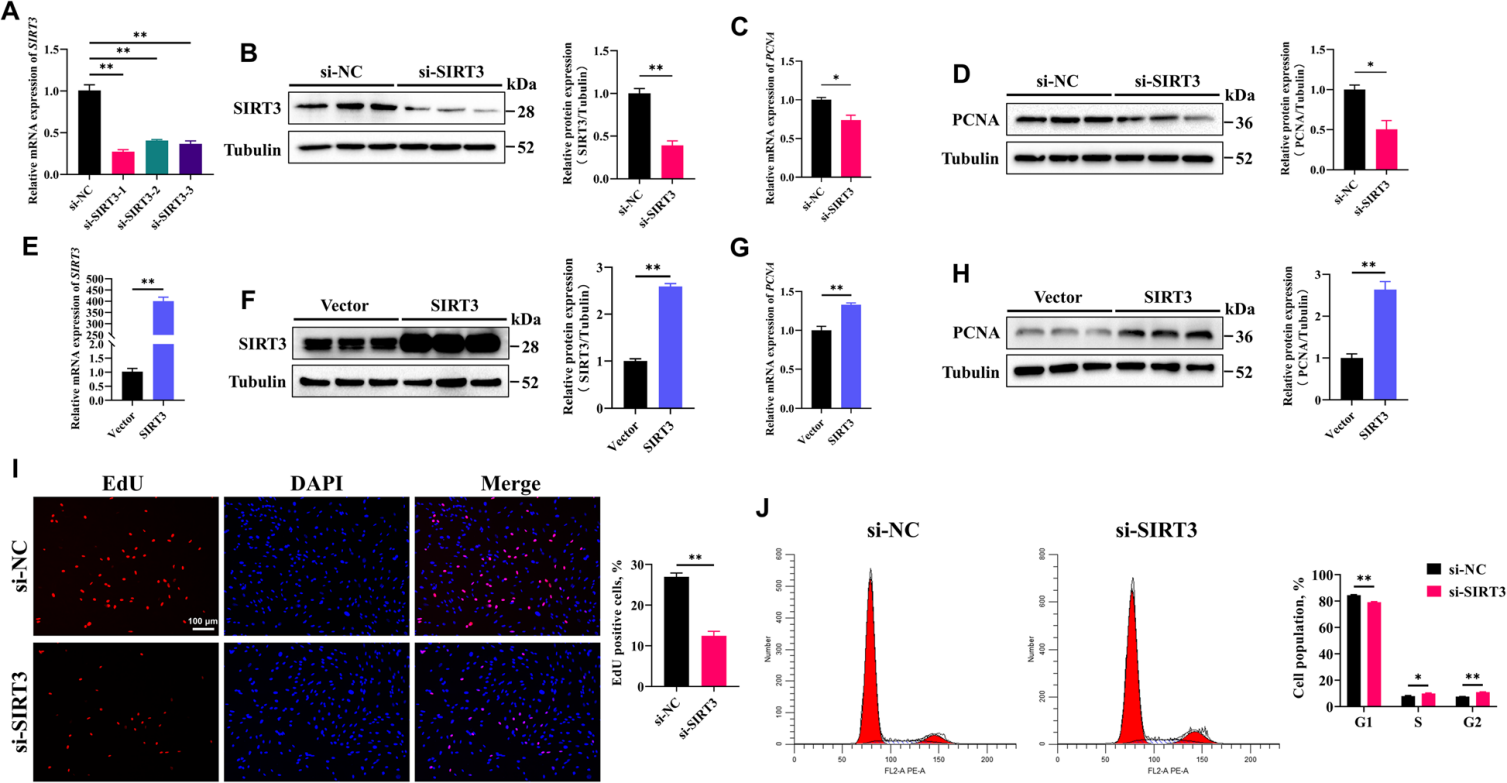
SIRT3 promotes the proliferation of GCs. **A** and **B** *SIRT3* knockdown efficiency was confirmed after transfection with si**-**SIRT3 compared with si**-**NC for 48 h. **C** and **D** Relative mRNA and protein expression levels of PCNA in GCs following *SIRT3* knockdown. **E** and **F** *SIRT3 *overexpression efficiency was confirmed after transfection with the overexpression plasmid for 48 h. **G** and **H** Relative mRNA and protein expression levels of PCNA in GCs following *SIRT3* overexpression. **I** EdU staining assay of GCs following *SIRT3* knockdown. Positive cells were stained with EdU in red, and cell nuclei were dyed with DAPI in blue; scale bar = 100 μm. **J** The cell cycle distribution of GCs was analyzed by flow cytometry. The data are presented as the mean ± SEM of three independent experiments. Statistical significance was determined using Student’s *t*-test, ^*^P < 0.05, ^**^P < 0.01

Follicular development and maturation depend on the proliferation of GCs. To investigate the effect of SIRT3 on GC proliferation, we constructed three pairs of siRNAs targeting the goat *SIRT3* gene and selected the most efficient pair. After transfection with siRNA, both the mRNA and protein expression levels of SIRT3 were significantly reduced (Fig. 3A and B). Correspondingly, the mRNA and protein levels of PCNA were also significantly decreased (Fig. 3C and D). We also constructed a plasmid for *SIRT3* overexpression to further assess its effects. Following transfection with the *SIRT3* overexpression plasmid, both the mRNA and protein levels of SIRT3 (Fig. 3E and F) and PCNA were significantly increased (Fig. 3G and H). In terms of cell phenotype, silencing *SIRT3* expression reduced the number of EdU**-**positive cells (Fig. 3I). Additionally, flow cytometry analysis revealed that *SIRT3* knockdown resulted in a reduced proportion of GCs in the G1 phase, with a corresponding increases in the S and G2 phases (Fig. 3J).

**Fig. 4:**
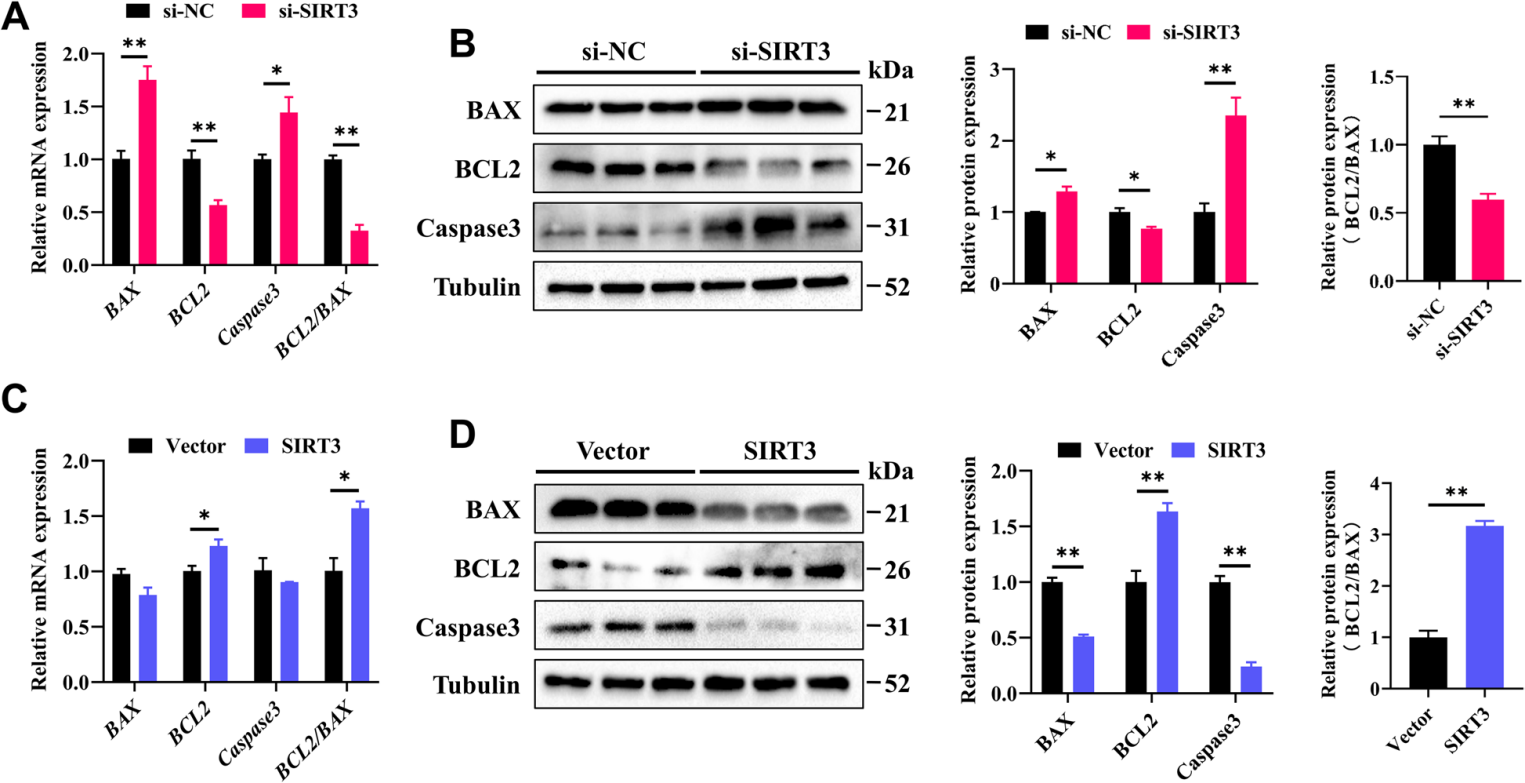
SIRT3 inhibits the apoptosis of GCs. **A** The mRNA expression levels of *BAX*, *BCL2*, and *Caspase3* in GCs following *SIRT3* knockdown. **B** The protein expression levels of BAX, BCL2, and Caspase3 in GCs were analyzed by Western blotting following *SIRT3* knockdown. **C** The mRNA expression of *BAX*, *BCL2*, and *Caspase3* in GCs following *SIRT3* overexpression. **D** The protein expression levels of BAX, BCL2, and Caspase3 in GCs were analyzed by Western blotting following *SIRT3* overexpression. The data are presented as the mean ± SEM of three independent experiments. Statistical significance was determined using Student’s *t*-test, ^*^P < 0.05, ^**^P < 0.01

We further examined the effect of SIRT3 on the expression of apoptosis**-**related genes in GCs. The results revealed that *SIRT3* knockdown significantly increased the mRNA and protein levels of BAX and Caspase3, whereas the expression of BCL2 and the BCL2/BAX ratio were significantly decreased (Fig. 4A and B). In contrast, overexpression of SIRT3 led to decreases in the protein levels of BAX and Caspase3, despite no significant changes in their mRNA levels, whereas both the mRNA and protein expression levels of BCL2 and the BCL2/BAX ratio were significantly increased (Fig. 4C and D). These findings indicate that SIRT3 promotes the proliferation of GCs and inhibits their apoptosis.

### SIRT3 enhances mitochondrial function in GCs

**Fig. 5:**
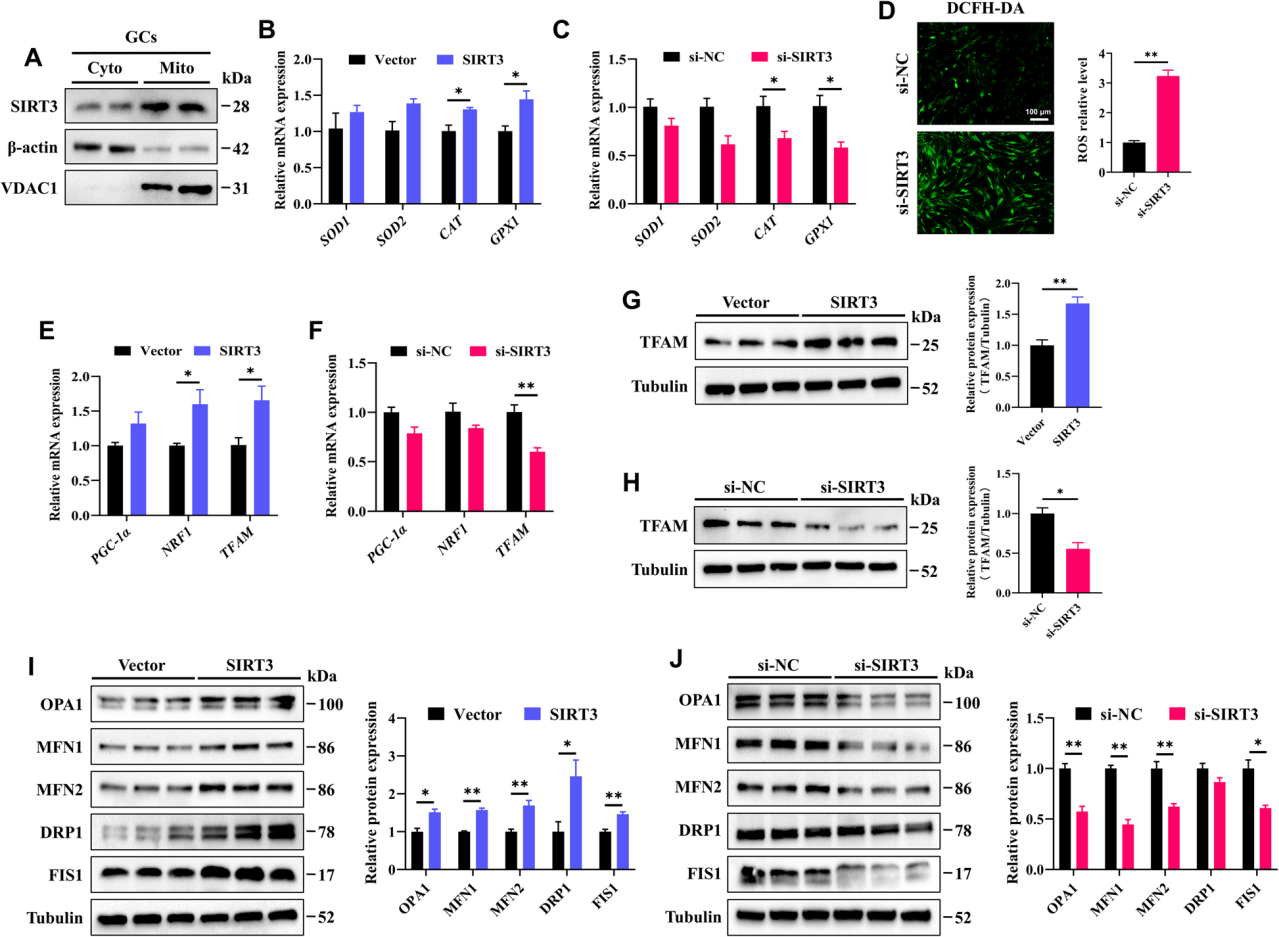
SIRT3 enhances mitochondrial function in GCs. **A** Expression and localization of the SIRT3 protein in the cytoplasm (Cyto) and mitochondria (Mito) of GCs. **B **and** C** The mRNA expression levels of oxidative stress**-**related genes (*SOD1*, *SOD2*, *CAT*, and *GPX1)* were quantified by RT-qPCR. **D** Total ROS levels in GCs transfected with si**-**NC or si**-**SIRT3 were measured using DCFH**-**DA; scale bar = 100 μm. **E **and** F** The mRNA levels of mitochondrial biogenesis-related genes (*PGC-1α*, *NRF1*, and *TFAM*) were quantified by RT-qPCR. **G **and** H** Relative expression of the TFAM protein in GCs following different treatments. **I **and** J** Relative expression levels of mitochondrial dynamics**-**related proteins (OPA1, MFN1, MFN2, DRP1, and FIS1) in GCs following different treatments. The data are presented as the mean ± SEM of three independent experiments. Statistical significance was determined using Student’s *t*-test, ^*^P < 0.05, ^**^P < 0.01

SIRT3 is a key enzyme that is predominantly localized in the mitochondria, where it regulates mitochondrial function and metabolism. First, we confirmed the subcellular distribution of SIRT3 in GCs, observing its primary expression in the mitochondria, with a smaller fraction present in the cytoplasm (Fig. 5A). We then evaluated the effect of SIRT3 on the expression of oxidative stress**-**related genes (*SOD1*, *SOD2*, *CAT*, and *GPX1*) in GCs. The results revealed that the overexpression of SIRT3 significantly increased the mRNA levels of *CAT* and *GPX1*, and the mRNA levels of *SOD1* and *SOD2* also tended to increase, although the differences were not statistically significant (Fig. 5B). In contrast, downregulation of SIRT3 resulted in a significant decreases in the mRNA levels of *CAT* and *GPX1* (Fig. 5C). Moreover, reduced SIRT3 expression led to an increase in ROS production in GCs (Fig. 5D).

We next examined the effect of SIRT3 on the expression of mitochondrial biogenesis**-**related genes (*PGC-1α*, *NRF1*, and *TFAM*) in GCs. The results revealed that overexpression of SIRT3 significantly elevated both the mRNA and protein expression levels of TFAM (Fig. 5E and G), whereas *SIRT3* knockdown resulted in decreases in both its mRNA and protein expression (Fig. 5F and H). Consistent with these findings, *SIRT3* overexpression led to significant increases in the protein expression levels of mitochondrial fusion**-**related proteins (OPA1, MFN1, and MFN2) and fission**-**related proteins (DRP1 and FIS1) (Fig. 5I). In contrast, *SIRT3* downregulation caused the opposite effects, with the exception of DRP1, whose expression did not significantly change (Fig. 5J). Together, these results confirmed that *SIRT3* overexpression enhances mitochondrial function in GCs.

### The SIRT3 protein interacts with the CPT2 protein

**Fig. 6:**
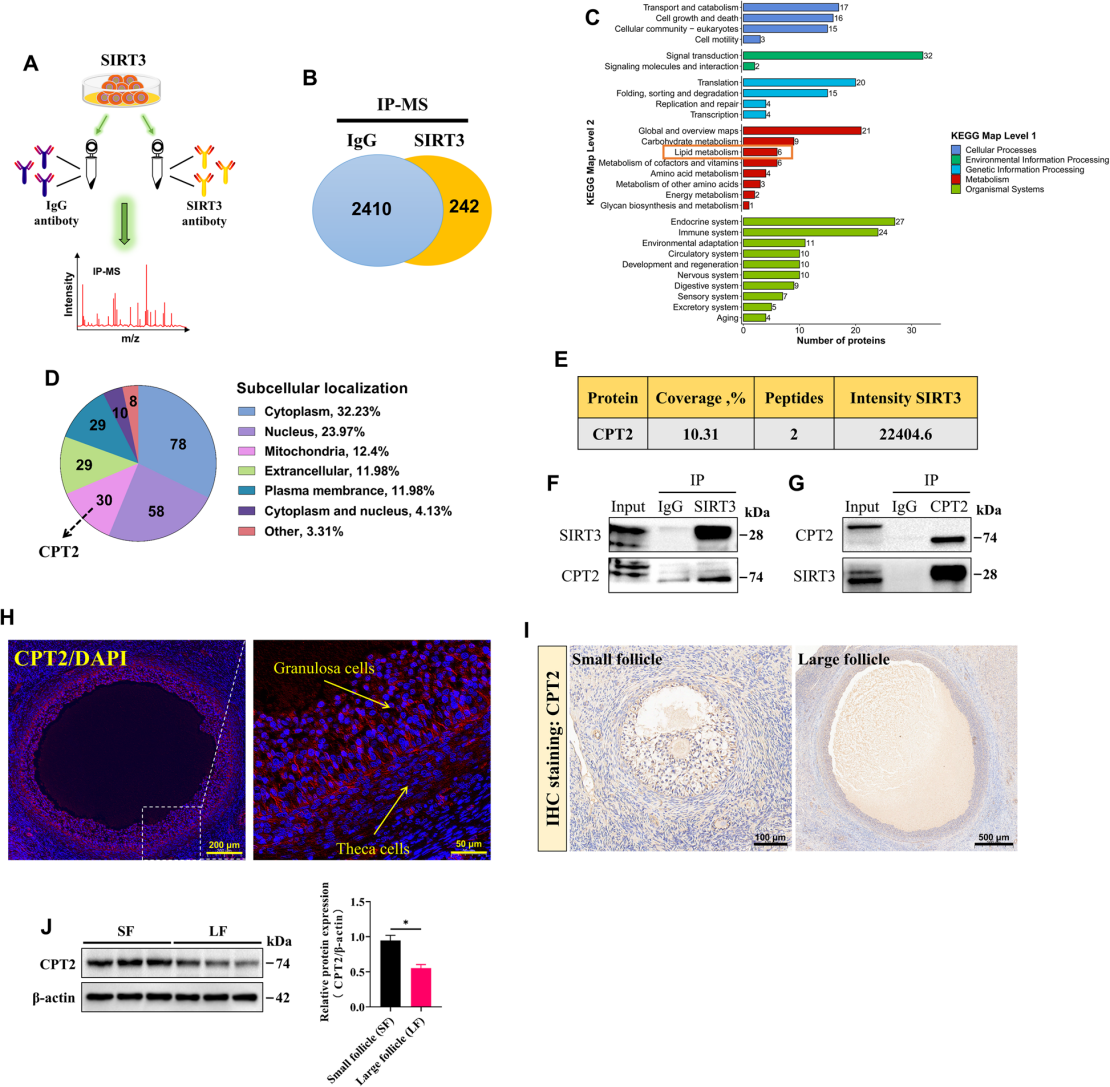
SIRT3 protein interacts with the CPT2 protein. **A** Overview of IP‒MS analysis of GCs (*n* = 1). **B** Proteins were identified by coimmunoprecipitation with SIRT3 and IgG antibodies in GCs. **C** KEGG pathway analysis of the identified proteins. **D** Subcellular localization analysis of the identified proteins. **E** Intensity of the interaction between SIRT3 and CPT2 in the IP‒MS. **F **and** G** Co-IP assay was used to reveal the interaction between SIRT3 and CPT2. **H** Representative images of immunofluorescence staining for CPT2 in goat ovaries; scale bar = 200 μm (left) and 50 μm (right). **I** Representative images of immunohistochemical staining for CPT2 in goat ovaries; scale bar = 100 μm (left) and 500 μm (right). **J** The protein expression levels of CPT2 in GCs from small follicles (SF) and large follicles (LF) were analyzed by Western blotting. The data are presented as the mean ± SEM of three independent experiments. Statistical significance was determined using Student’s *t*-test, ^*^P < 0.05

To elucidate the potential mechanisms and targets by which SIRT3 regulates the function of GCs, we performed IP‒MS analysis to identify downstream proteins that interact with SIRT3 in GCs (Fig. 6A). After 2,410 proteins were removed from the IP‒IgG eluate, 242 proteins that specifically interacted with SIRT3 were identified (Fig. 6B and Table S3). KEGG pathway classification analysis of these identified proteins revealed that 52 proteins were involved in metabolism, including lipid metabolism (Fig. 6C and Table S4). In addition, subcellular localization analysis revealed that 30 of these proteins were localized to the mitochondria, including CPT2, a key enzyme in fatty acid β**-**oxidation (FAO), which interacted with SIRT3 (Fig. 6D and E, and Table S5). Importantly, the Co**-**IP assay results also demonstrated that SIRT3 and CPT2 could pull down one another, further confirming that CPT2 is an interacting protein of SIRT3 (Fig. 6F and G). To investigate the function of CPT2, we examined the localization and expression of CPT2 in goat ovarian follicles. Immunofluorescence staining of ovarian tissue revealed that CPT2 was expressed predominantly in GCs within the follicles (Fig. 6H). Notably, both immunohistochemistry and Western blot analyses revealed that CPT2 expression was significantly greater in GCs from small follicles than in those from large follicles (Fig. 6I and J). These findings suggest that CPT2 may be regulated by SIRT3 and play a critical role in GCs.

### SIRT3 mediates the delactylation of CPT2

**Fig. 7:**
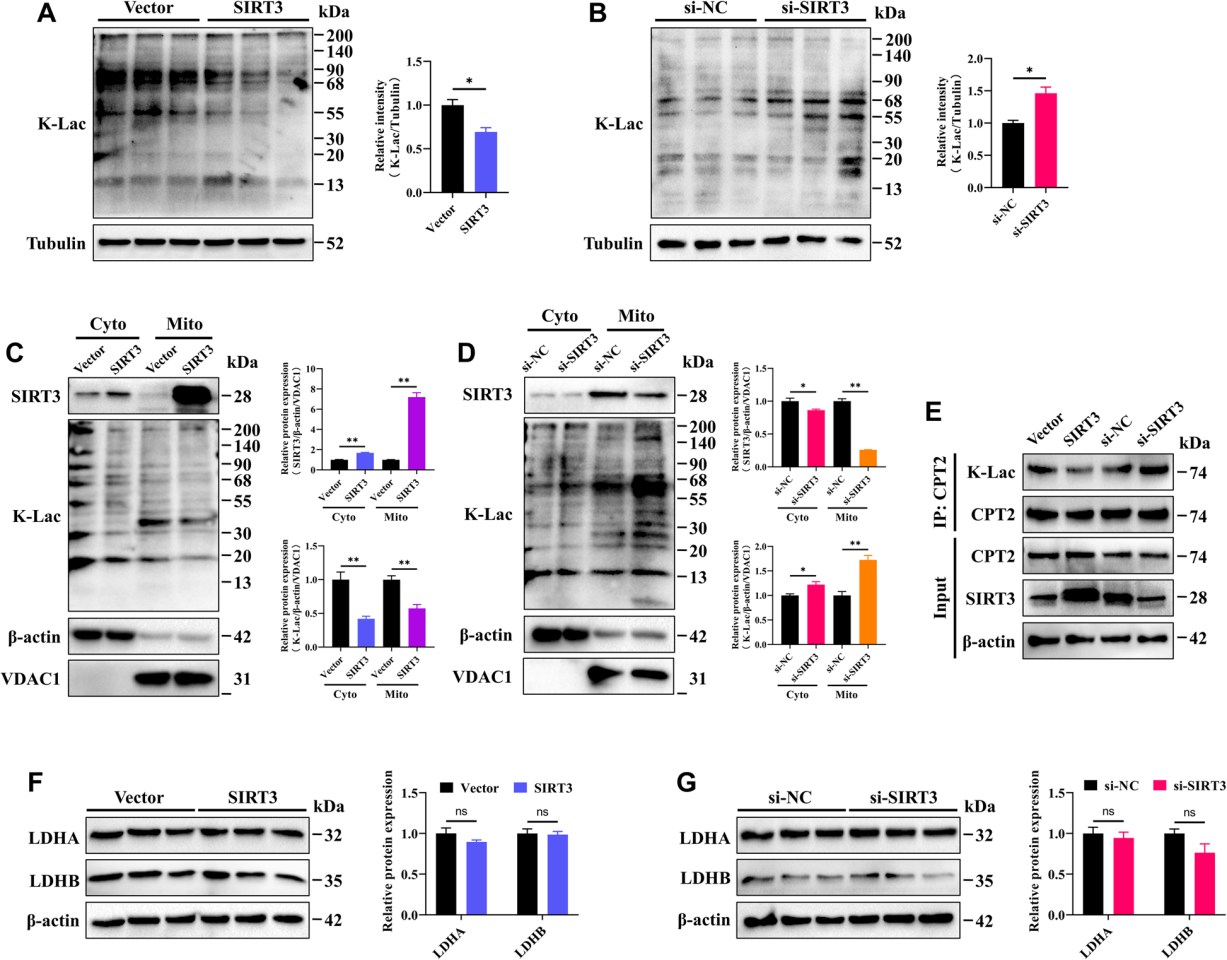
SIRT3 mediates the delactylation of CPT2. **A** The levels of K**-**Lac in GCs following SIRT3 overexpression were quantified. **B** The levels of K**-**Lac in GCs following SIRT3 knockdown. **C** The levels of K**-**Lac in the cytoplasm (Cyto) and mitochondria (Mito) of GCs following SIRT3 overexpression. **D** The levels of K**-**Lac in the Cyto and Mito of GCs following SIRT3 knockdown. **E** Immunoprecipitation of protein extracts from different treatment groups was performed with an anti-CPT2 antibody, followed by immunoblot analysis with an anti**-**K**-**Lac antibody. **F **and** G** Relative protein expression levels of LDHA and LDHB in GCs after different treatments. The data are presented as the mean ± SEM of three independent experiments. Statistical significance was determined using Student’s *t*-test, ^*^P < 0.05, ^**^P < 0.01, ns, not significant

Given the significant impact of PTMs on protein function and the role of SIRT3 as an "eraser" of various PTMs, we investigated whether SIRT3 influences the K**-**Lac of CPT2, a novel and widely distributed PTM. We found that the overexpression of SIRT3 resulted in a significant decrease in the global K**-**Lac level in GCs, whereas the silencing of SIRT3 had the opposite effect (Fig. 7A and B). At the subcellular level, we further explored the specific impact of SIRT3 on the K**-**Lac levels of cytoplasmic and mitochondrial proteins in GCs. The results showed that SIRT3 overexpression induced a significant decreases in K**-**Lac levels in both cytoplasmic and mitochondrial proteins in GCs, whereas silencing SIRT3 increased K**-**Lac levels in mainly mitochondrial proteins (Fig. 7C and D). As expected, the immunoprecipitation results revealed that the overexpression of SIRT3 led to a decrease in the K**-**Lac level of CPT2, whereas the knockdown of SIRT3 increased it (Fig. 7E). Additionally, we found that the delactylation of CPT2 by SIRT3 in GCs is likely a direct effect, as changes in SIRT3 expression did not alter the protein levels of LDHA and LDHB, two enzymes closely involved in lactate production, which serve as the important substrates for protein lactylation (Fig. 7F and G).

### SIRT3 enhances the stability of the CPT2 protein

**Fig. 8:**
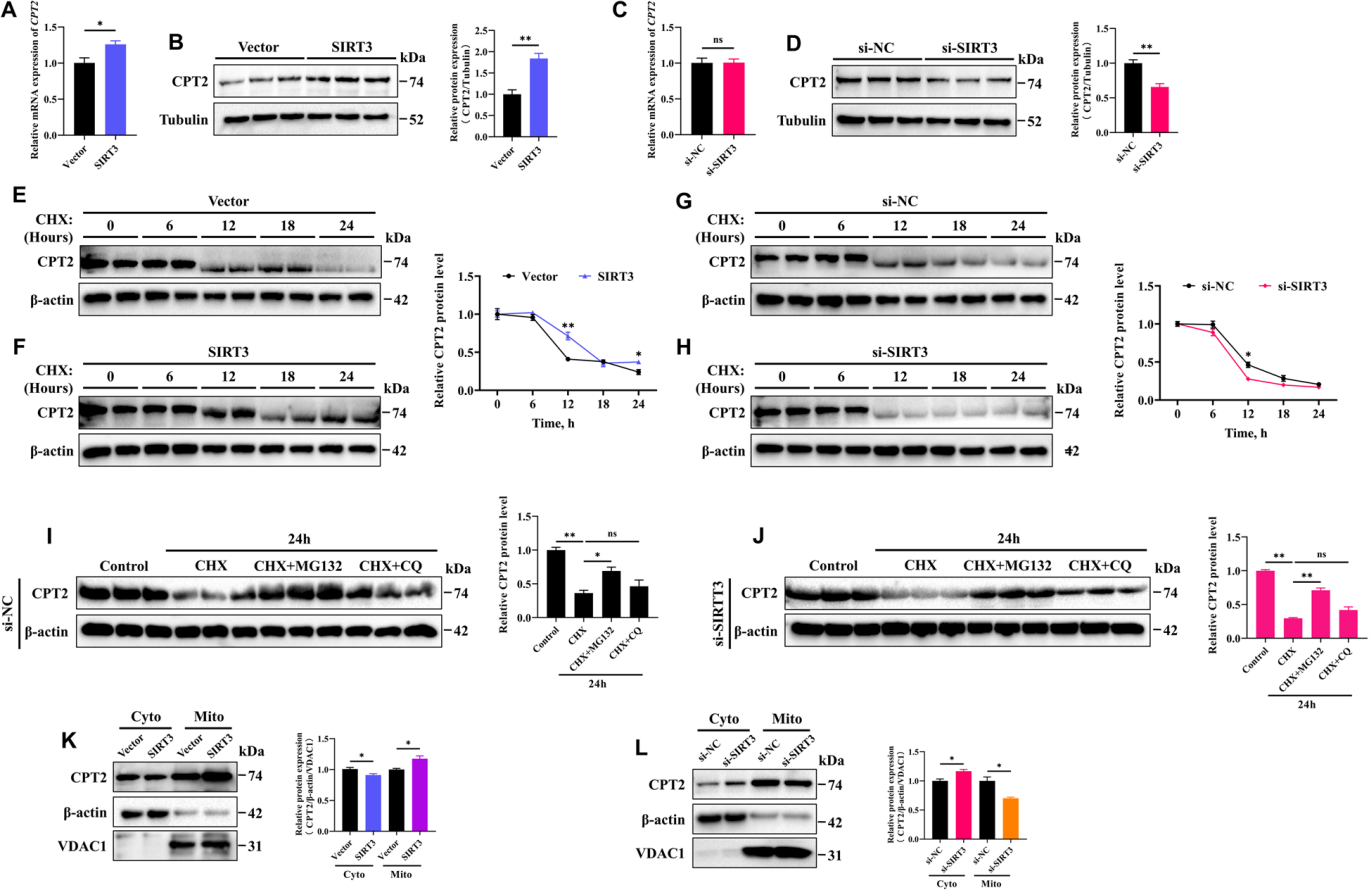
SIRT3 enhances the stability of the CPT2 protein. **A** and** B** Relative mRNA and protein expression levels of CPT2 in GCs following SIRT3 overexpression. **C **and** D** Relative mRNA and protein levels of CPT2 in GCs following SIRT3 knockdown. **E **and** F** The protein expression levels of CPT2 were analyzed in GCs treated with vector or SIRT3 overexpression, followed by treatment with 20 µg/mL cycloheximide (CHX) for the indicated times. **G **and** H** The protein expression levels of CPT2 were analyzed in GCs treated with si**-**NC or si**-**SIRT3, followed by treatment with 20 µg/mL CHX for the indicated times. **I **and** J** CHX (20 µg/mL) was added to the GCs following transfection with si**-**NC and si**-**SIRT3, and CQ (30 µmol/L) or MG132 (20 µmol/L) was added simultaneously for 24 h. The expression of CPT2 was analyzed by Western blotting. **K **and** L** The protein expression and localization of CPT2 in the cytoplasm (Cyto) and mitochondria (Mito) in GCs following SIRT3 overexpression or knockdown. The data are presented as the mean ± SEM of three independent experiments. Statistical significance was determined using Student’s *t*-test, ^*^P < 0.05, ^**^P < 0.01, ns, not significant

To further elucidate the specific changes in the CPT2 protein caused by SIRT3, we first examined its effect on CPT2 expression. The results revealed that SIRT3 overexpression significantly increased *CPT2* mRNA levels, but the increase in CPT2 protein levels was more significant (Fig. 8A and B). In contrast, silencing SIRT3 led to a significant decrease in CPT2 protein levels, although it had no effect on *CPT2* mRNA levels (Fig. 8C and D), which showed that SIRT3 primarily regulates CPT2 at the protein level. Next, to investigate the impact of SIRT3 on CPT2 protein stability, we performed cycloheximide (CHX) chase assays. Compared with that in the control group, the half**-**life of the CPT2 protein was longer in the SIRT3**-**overexpressing GCs (Fig. 8E and F). Conversely, CPT2 degradation was significantly accelerated in SIRT3**-**knockdown GCs (Fig. 8G and H). These findings suggest that SIRT3 enhances the stability of the CPT2 protein. We then explored the degradation pathway of CPT2. After treatment with CHX for 24 h, CPT2 protein levels were significantly reduced in both the control and SIRT3**-**knockdown GCs. However, this reduction was reversed by the proteasome inhibitor MG132 but not by the lysosomal inhibitor chloroquine (CQ) (Fig. 8I and J). Interestingly, at the subcellular level, overexpression of SIRT3 increased CPT2 protein levels in the mitochondria, whereas its expression in the cytoplasm was reduced (Fig. 8K). In contrast, silencing SIRT3 had the opposite effect (Fig. 8L). These results suggest that SIRT3 may also influence the subcellular localization of CPT2, thereby regulating its function in GCs.

### CPT2 enhances mitochondrial FAO and dynamics in GCs

**Fig. 9:**
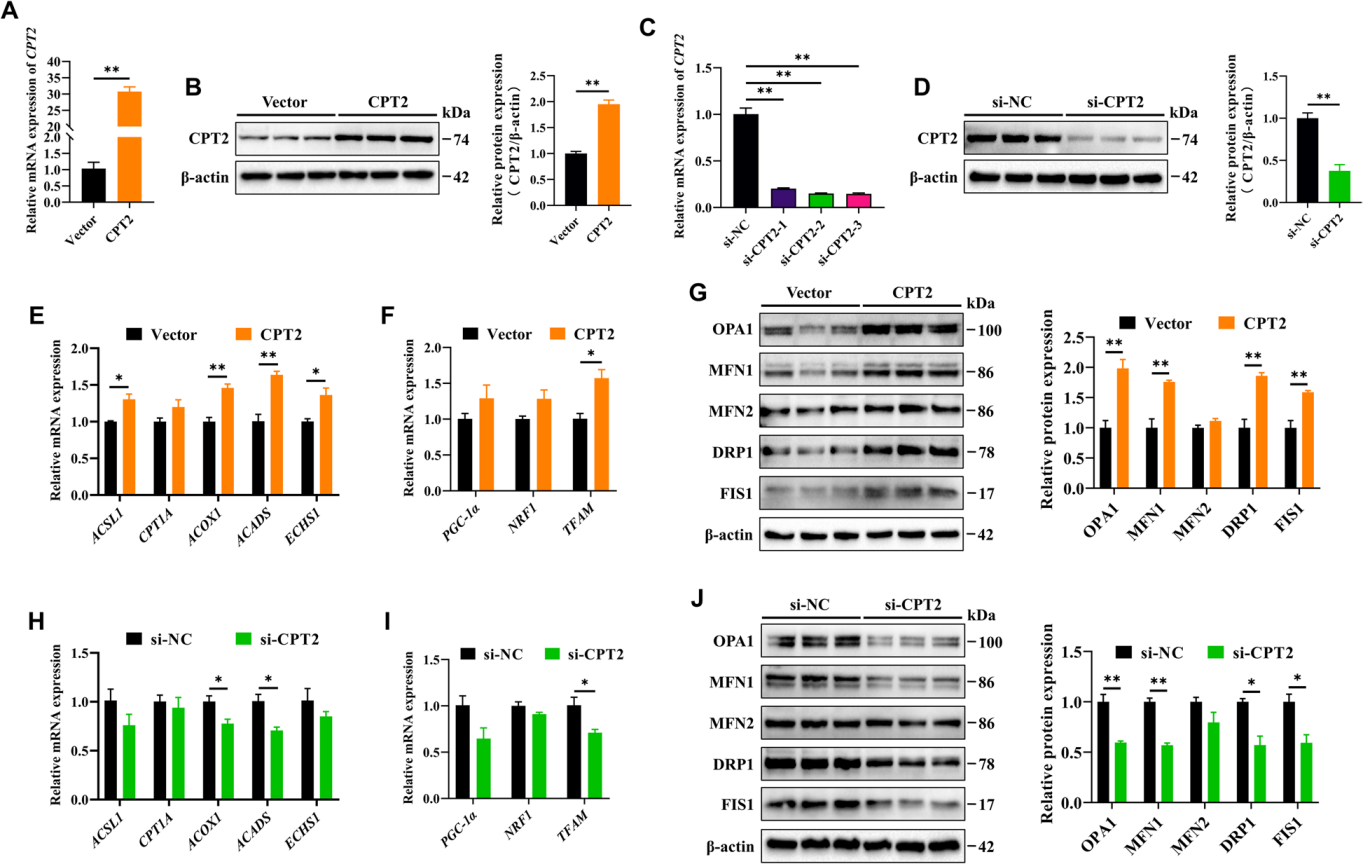
CPT2 enhances mitochondrial FAO and dynamics in GCs. **A **and** B** The overexpression efficiency of CPT2 was confirmed following transfection for 48 h. **C **and** D** The efficiency of CPT2 inhibition was confirmed following transfection with si**-**CPT2 or si**-**NC for 48 h. **E** The mRNA expression levels of FAO**-**related genes, including *ACSL1*, *CPT1A*, *ACOX1*, *ACADS*, and *ECHS1*, were detected by RT-qPCR in GCs after overexpression of CPT2. **F** The mRNA levels of *PGC-1α*, *NRF1*, and *TFAM* in GCs following *CPT2* overexpression. **G** Relative protein levels of OPA1, MFN1, MFN2, DRP1, and FIS1 in GCs after overexpression of CPT2. **H** The mRNA levels of FAO**-**related genes in GCs following *CPT2* knockdown. **I** The mRNA levels of *PGC-1α*, *NRF1*, and *TFAM* in GCs following *CPT2* knockdown. **J** Relative protein levels of OPA1, MFN1, MFN2, DRP1, and FIS1 in GCs following CPT2 knockdown. The data are presented as the mean ± SEM of three independent experiments. Statistical significance was determined using Student’s *t*-test, ^*^P < 0.05, ^**^P < 0.01

Mitochondria are central to FAO; thus, we hypothesized that CPT2 may affect mitochondrial function by regulating FAO in GCs. To test this hypothesis, we synthesized *CPT2* overexpression plasmids (Fig. 9A and B) and siRNAs (Fig. 9C and D) and successfully verified their efficiency. First, we confirmed the effect of CPT2 on FAO in GCs by evaluating the expression levels of FAO**-**related genes (*ACSL1*, *CPT1A*, *ACOX1*, *ACADS*, and *ECHS1*) via RT-qPCR analysis. Compared with the vector, *CPT2* overexpression significantly increased the mRNA levels of *ACSL1*, *ACOX1*, *ACADS*, and *ECHS1*, whereas no significant change was observed for *CPT1A* (Fig. 9E). Additionally, CPT2 overexpression significantly increased the mRNA level of *TFAM* and the protein levels of OPA1, MFN1, DRP1, and FIS1 (Fig. 9F and G). In contrast, *CPT2* knockdown significantly reduced the mRNA levels of *ACOX1*, *ACADS*, and *TFAM*, as well as the protein levels of OPA1, MFN1, DRP1, and FIS1 (Fig. 9H–J). These results suggest that high expression of CPT2 enhances mitochondrial FAO and dynamics in GCs.

### CPT2 promotes GC proliferation via the β-catenin/CCND1pathway

**Fig. 10:**
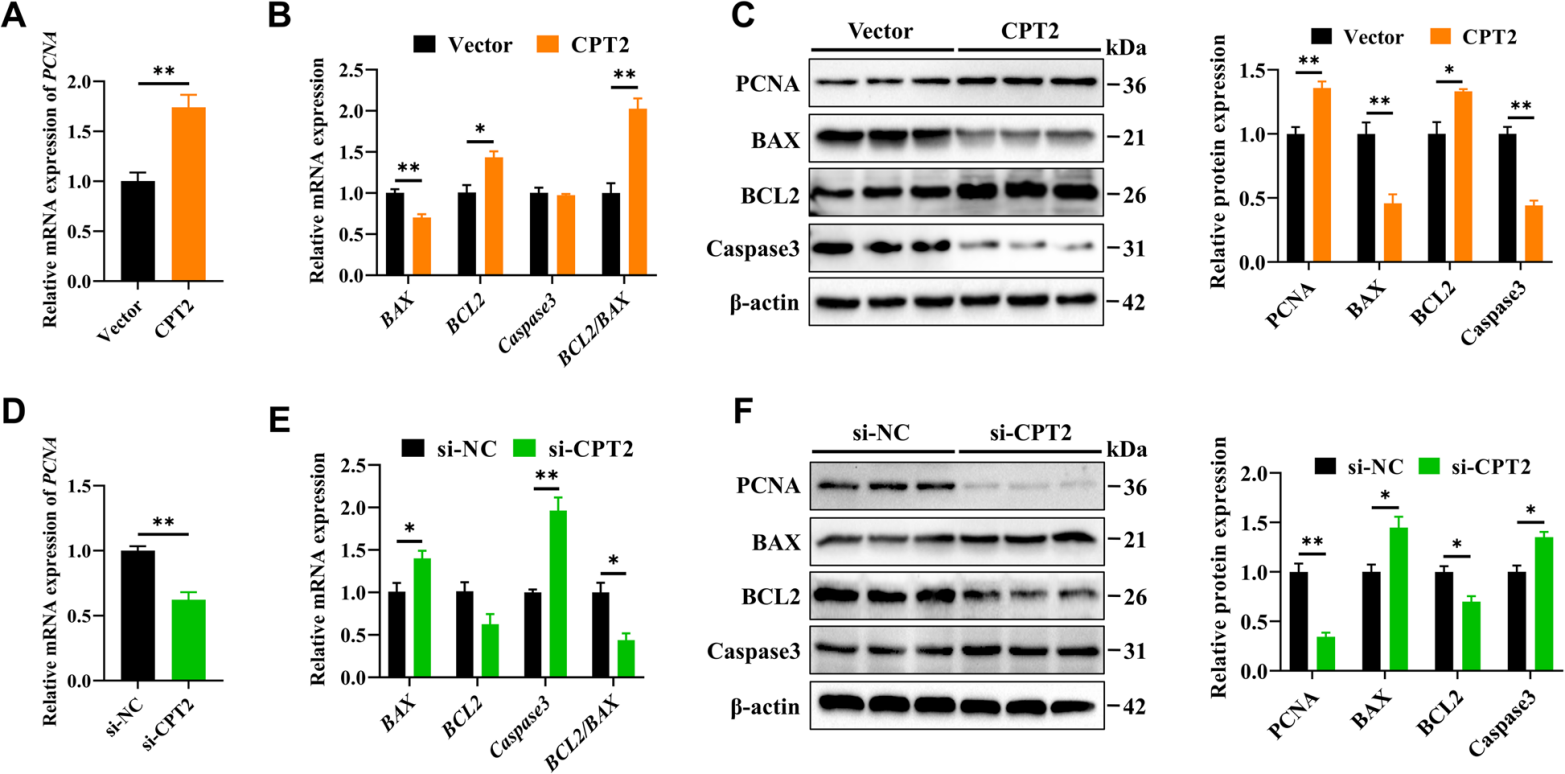
CPT2 promotes the proliferation of GCs. **A** The mRNA levels of *PCNA* in GCs following *CPT2 *overexpression. **B** The mRNA levels of *BAX*, *BCL2*, and *Caspase3* in GCs following *CPT2* overexpression. **C** The protein levels of PCNA, BAX, BCL2, and Caspase3 in GCs following CPT2 overexpression. **D** The mRNA levels of *PCNA* in GCs following *CPT2* knockdown. **E** The mRNA levels of *BAX*, *BCL2*, and *Caspase3* in GCs following *CPT2* knockdown. **F** The protein levels of PCNA, BAX, BCL2, and Caspase3 in GCs following CPT2 knockdown. The data are presented as the mean ± SEM of three independent experiments. Statistical significance was determined using Student’s *t*-test, ^*^P < 0.05, ^**^P < 0.01

We next investigated the effect of CPT2 on GC proliferation. The results revealed that, upon *CPT2* overexpression, both the mRNA and protein expression levels of PCNA and BCL2 were significantly increased in GCs, whereas the mRNA and protein expression levels of BAX were significantly decreased. Notably, Caspase3 expression was significantly reduced only at the protein level (Fig. 10A–C). These changes were reversed when *CPT2* was knocked down, as evidenced by decreased expression of PCNA and BCL2 and increased expression of BAX and Caspase3 (Fig. 10D–F).

**Fig. 11:**
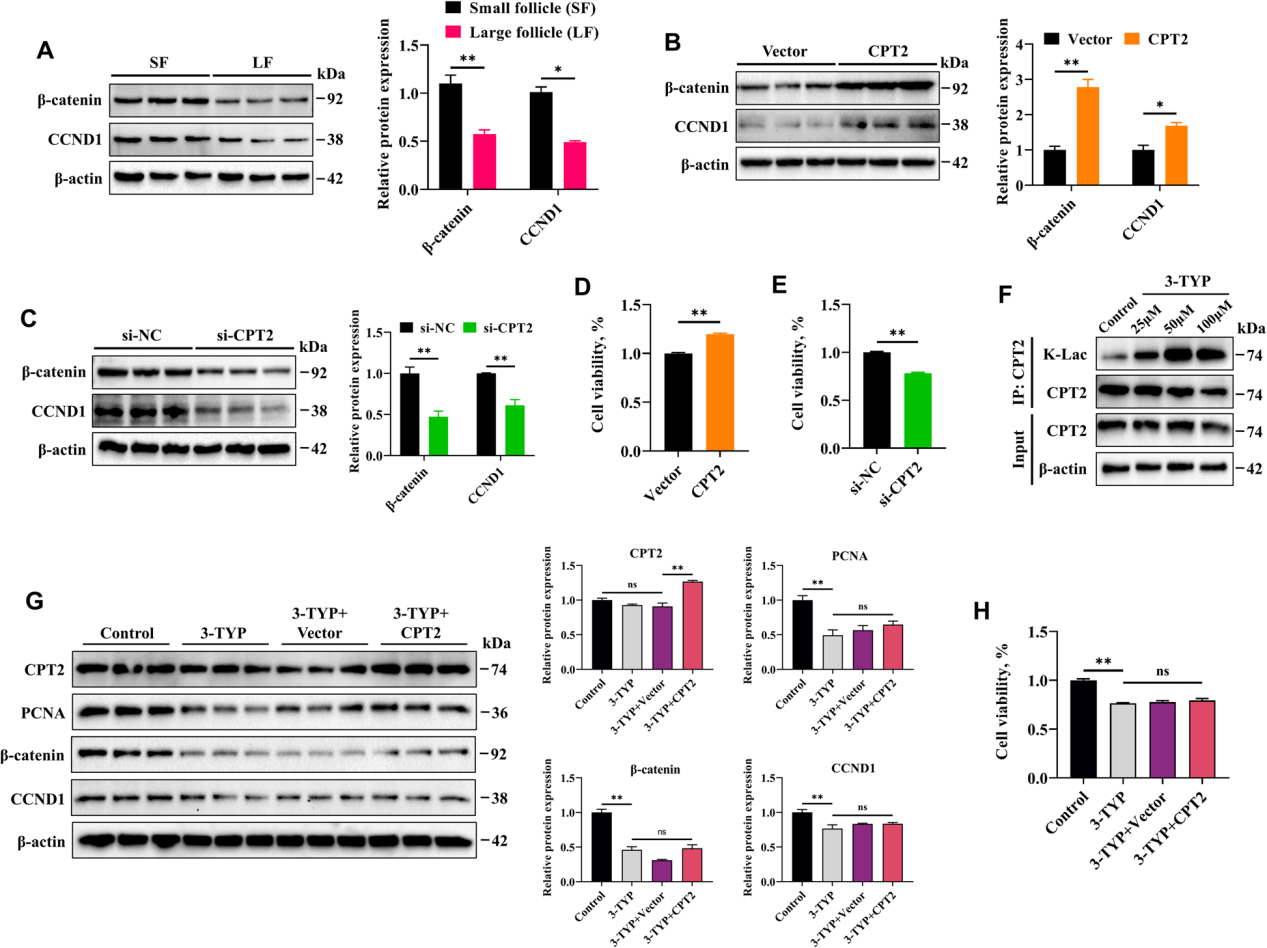
CPT2 promotes GC proliferation via the β**-**catenin/CCND1 pathway. **A** The protein levels of β**-**catenin and CCND1 in GCs from small follicles (SF) and large follicles (LF) were analyzed by Western blotting. **B** The protein levels of β**-**catenin and CCND1 in GCs following CPT2 overexpression were analyzed by Western blotting. **C** The protein levels of β**-**catenin and CCND1 in GCs following CPT2 knockdown were analyzed by Western blotting. **D** and **E** CCK**-**8 assay for detecting cell viability. **F** Immunoprecipitation was used to analyze the effects of different concentrations of 3**-**TYP on the K**-**Lac level of CPT2. **G** Relative protein expression levels of CPT2, PCNA, β**-**catenin, and CCND1 in GCs following different treatments. **H** CCK**-**8 assay for detecting cell viability following different treatments. The data are presented as the mean ± SEM of three independent experiments. Statistical significance was determined using Student’s *t*-test, ^*^P < 0.05, ^**^P < 0.01

Previous studies have shown that the WNT/β**-**catenin pathway plays important roles in cell proliferation and follicular development and is potentially regulated by CPT2 [25, 26]. To investigate this further, we assessed the effectiveness of the β**-**catenin pathway in this study. The results indicated that β**-**catenin and its target protein CCND1 were expressed at significantly higher levels in GCs from small follicles (Fig. 11A), which supports the previous finding that GCs from small follicles rapidly proliferate. Similarly, overexpression of CPT2 significantly increased the protein levels of both β**-**catenin and CCND1 (Fig. 11B). In contrast, knockdown of CPT2 led to reduced expression of β**-**catenin and CCND1 (Fig. 11C). Furthermore, CCK-8 assays revealed that *CPT2* overexpression significantly increased GC viability, whereas interference with CPT2 had the opposite effect (Fig. 11D and E). These results suggest that CPT2 can indeed promote GC proliferation via the β**-**catenin/CCND1 pathway. Finally, the use of 3-TYP (50 µmol/L), an inhibitor of SIRT3, increased the K-Lac level of CPT2 (Fig. 11F) and counteracted the proliferative effects of CPT2 overexpression on GCs, including decreased PCNA, β**-**catenin, and CCND1 protein levels and reduced cell viability (Fig. 11G and H). These results suggest that SIRT3 enhances mitochondrial function in GCs by increasing CPT2 protein expression and that CPT2 also promotes GC proliferation by upregulating β**-**catenin and CCND1, thereby supporting follicular development.

## Discussion

Improving reproductive performance has always been a key objective in goat farming. The ovary, as an important reproductive organ of female mammals, is closely related to goat reproduction. Increasing ovarian function, particularly follicular development, is crucial for improving goat fertility. The development of follicles from the primordial stage to the mature stage, followed by ovulation, is a tightly regulated process [27]. Therefore, ensuring the proper development of follicles lays the foundation for increasing the ovulation rate and the number of lambs per litter. Previous studies have indicated that *SIRT3* is a potential candidate gene affecting reproductive traits in goats [28]. In particular, SIRT3 plays a significant role in regulating GC function and the ovarian reserve in female humans and bovines [21, 29]. However, whether SIRT3 influences the follicular development process in goats and the underlying mechanisms remain unclear. Our study revealed that SIRT3 stabilizes the CPT2 protein through delactylation, thereby improving mitochondrial function and promoting GC proliferation via the β**-**catenin/CCND1 pathway in goats. These findings may provide new insights into the molecular mechanisms regulating follicular development.

GC proliferation is essential for follicular growth and development, whereas GC apoptosis leads to follicular atresia [30, 31]. Previous studies have shown that during follicular development in pigs, the proliferative capacity of GCs is significantly greater in small follicles than in large follicles, with GCs from large follicles exhibiting a greater rate of apoptosis [32]. Similarly, the results of this study demonstrate that, in goats, the mRNA and protein expression levels of the cell proliferation marker PCNA are significantly greater in GCs from small follicles than in GCs from large follicles, whereas the apoptosis**-**related proteins BAX and Caspase3 are relatively more highly expressed in GCs from large follicles. These findings suggest that the rapid proliferation of GCs from small follicles may be a conserved phenomenon during mammalian follicular development. However, GC proliferation is regulated by a complex network of factors. The role of sirtuins in influencing various cellular fates has been well established [33, 34], and each family member has a relatively unique biological role. In this study, we observed that SIRT3 was highly expressed in GCs from small follicles. In vitro, SIRT3 overexpression promoted GC proliferation and inhibited apoptosis, which is consistent with its reported effects in other human and mouse cell types [35–37]. These results highlight the important role of SIRT3 in regulating GC function and provide further evidence for its possible involvement in follicular development.

As a mitochondrial protein, SIRT3 plays a critical role in maintaining mitochondrial quality and homeostasis, including redox balance [38], mitochondrial dynamics [39], and energy metabolism [40]. Notably, the function of GCs is closely associated with mitochondrial quality. For example, SIRT3 can alleviate β**-**hydroxybutyrate**-**induced mitochondrial dysfunction in bovine ovarian GCs, thereby improving cellular damage in GCs [21]. Similarly, the absence of SIRT3 in human ovarian GCs leads to mitochondrial dysfunction and elevated oxidative stress, resulting in glucose metabolism defects [41]. Consistent with these findings, in the present study, SIRT3 overexpression promoted the expression of genes or proteins related to mitochondrial biogenesis (TFAM) and mitochondrial dynamics (OPA1, MFN1, MFN2, DRP1, and FIS1), thereby enhancing mitochondrial function in goat GCs. Conversely, SIRT3 deficiency disrupted mitochondrial dynamics, impairing GC function. Importantly, a previous study reported that SIRT3 deficiency leads to a uniform decrease in the levels of proteins involved in mitochondrial dynamics and their uneven mitochondrial distribution in the mouse ovaries, thereby accelerating follicle depletion and ovarian ageing [17], which is consistent with our current findings. These findings suggest that SIRT3 is a critical regulator of mitochondrial function, and may be able to modulate ovarian function and support follicular development by influencing the GC phenotype.

By investigating the mechanism by which SIRT3 regulates GC function, our study revealed that SIRT3 interacts with CPT2, a mitochondrial metabolic enzyme that plays a key role in FAO by catalyzing the conversion of fatty acyl**-**carnitine to fatty acyl**-**CoA. Interestingly, many studies have shown that CPT2 is the target of multiple PTMs. At present, CPT2 can undergo acetylation [42], succinylation [43], and lactylation [44] and is reversely regulated by related "erasers". In addition, depending on the type of modification, the stability and activity of the CPT2 protein also change accordingly. Notably, recent research has identified SIRT3 as a lysine delactylase that regulates protein stability and activity through delactylation [22]. In this study, we found that, compared to mRNA level, SIRT3 more strongly regulates CPT2 at the protein level. Based on these findings, we hypothesized that lactylation serves as the "bridge" by which SIRT3 regulates CPT2. To test this hypothesis, we conducted a series of experiments and observed that SIRT3 overexpression mediates the delactylation of total protein, mitochondrial protein, and specifically CPT2 in GCs. Additionally, SIRT3 modulates the degradation and subcellular localization of the CPT2 protein. Unfortunately, although previous studies have shown that increased levels of certain PTMs of the CPT2 protein led to its inactivation, whether the changes in CPT2 protein function caused by SIRT3 in GCs are specifically driven by lactylation and the lysine sites of SIRT3**-**mediated delactylation of CPT2 are still unclear and need further investigation.

Numerous studies have shown that the deficiency of CPT2 impairs mitochondrial FAO [45–47], and this metabolic dysregulation is often associated with mitochondrial dysfunction [48]. Conversely, increased CPT2 activity can enhance mitochondrial homeostasis by promoting FAO [49]. Consistent with these findings, our study demonstrated that CPT2 overexpression significantly enhanced FAO and mitochondrial dynamics in GCs, as evidenced by the increased expression levels of related key genes (*ACSL1*, *ACOX1*, *ACADS*, and *ECHS1*) and proteins (OPA1, MFN1, DRP1, and FIS1). In contrast, silencing CPT2 expression resulted in the opposite effect, further confirming the beneficial effects of CPT2 on mitochondria in GCs. Additionally, we observed that CPT2 overexpression promoted GC proliferation. We speculate that this effect is due to the sufficient energy provided by enhanced FAO for cell proliferation, as was previously confirmed in another study [50]. Regarding GC proliferation, previous studies have shown that the β**-**catenin pathway and its downstream target, CCND1, play significant roles in this process [51, 52]. Crucially, previous research has also indicated that CPT2 can regulate GC function through the β**-**catenin pathway [25]. In this study, we found that CPT2 overexpression significantly increased the expression of β**-**catenin and CCND1 in GCs. However, this effect was reversed by the SIRT3 inhibitor 3**-**TYP, suggesting that β**-**catenin/CCND1 may be a key pathway through which CPT2 regulates GC proliferation and that SIRT3 plays a critical role in this process. These findings collectively suggest that SIRT3 may be a key regulator of follicular development, influencing GC function by modulating the expression of downstream CPT2. This study provides valuable insights into the molecular mechanisms of follicular development and suggests that SIRT3 may be a potential therapeutic target for improving fertility in goats and other livestock with low fertility rates.

## Conclusions

In conclusion, we identified the beneficial effects of SIRT3 on the mitochondrial function and proliferation of goat GCs. Mechanistically, SIRT3 stabilizes its downstream interacting protein, CPT2, through delactylation, thereby promoting GC proliferation via the β**-**catenin-CCND1 pathway. This study not only contributes to the understanding of the mechanism by which SIRT3 regulates GC proliferation but also may provide new insights into the identification of molecular markers and regulatory pathways that drive follicle development.

## Supplementary Information

Additional file 1. Table S1. Primer information used for quantitative real**-**time PCR.

Additional file 2. Table S2. Antibodies used in this study.

Additional file 3. Table S3. Proteins identified by IP‒MS analysis.

Additional file 4. Table S4. KEGG classification results of 242 proteins identified by IP**-**SIRT3.

Additional file 5. Table S5. Subcellular localization results of 242 proteins identified by IP**-**SIRT3.

## Data Availability

The datasets used and/or analyzed during the current study are available from the corresponding author on reasonable request.

## Abbreviations

BSA: Bovine serum albumin
CCK**-**8: Cell counting kit**-**8
CHX: Cycloheximide
CPT2: Carnitine palmitoyl transferase 2
CQ: Chloroquine
EdU: 5**-**Ethynyl**-**2'**-**deoxyuridine
FAO: Fatty acid β**-**oxidation
FBS: Fetal bovine serum
GCs: Granulosa cells
IP‒MS: Immunoprecipitation coupled with mass spectrometry
K**-**Lac: Lysine lactylation
NCBI: National center for biotechnology information
OD: Optical density
PBS: Phosphate**-**buffered saline
PI: Propidium iodide
PTMs: Post**-**translational modifications
PVDF: Polyvinylidene fluoride
ROS: Reactive oxygen species
SDS**-**PAGE: Sodium dodecyl sulfate**–**polyacrylamide gel electrophoresis
siRNA: Small interfering RNA
SIRT3: Sirtuin 3
TBST: Tris-buffered saline containing 0.1% Tween**-**20

## References

[1] Chen W, Han Y, Chen Y, Liu X, Liang H, Wang C, et al. Potential candidate genes associated with litter size in goats: a review. Animals. 2025;15(1):82. 39795025 10.3390/ani15010082 PMC11718837

[2] Liu W, Chen C, Gao Y, Cui X, Zhang Y, Gu L, et al. Transcriptome dynamics and cell dialogs between oocytes and granulosa cells in mouse follicle development. Genom Proteom Bioinform. 2024;22(2):qzad001. 10.1093/gpbjnl/qzad001 PMC11423849 38955498

[3] Wang H, Huang Z, Shen X, Lee Y, Song X, Shu C, et al. Rejuvenation of aged oocyte through exposure to young follicular microenvironment. Nat Aging. 2024;4(9):1194–210. 39251866 10.1038/s43587-024-00697-x

[4] Khan S, Jamal MA, Khan IM, Ullah I, Jabbar A, Khan NM, et al. Factors affecting superovulation induction in goats (*Capra hericus*): an analysis of various approaches. Front Vet Sci. 2023;10:1152103. 37035816 10.3389/fvets.2023.1152103 PMC10079885

[5] Zhao Z, Zou X, Lu T, Deng M, Li Y, Guo Y, et al. Identification of mRNAs and lncRNAs involved in the regulation of follicle development in goat. Front Genet. 2020;11:589076. 33391342 10.3389/fgene.2020.589076 PMC7773919

[6] Wang X, Liao J, Shi H, Zhao Y, Ke W, Wu H, et al. Granulosa cell-layer stiffening prevents escape of mural granulosa cells from the post-ovulatory follicle. Adv Sci. 2024;11(33):e2403640. 10.1002/advs.202403640 PMC11434234 38946588

[7] Xu X, Jiang H, Wang D, Rehman SU, Li Z, Song X, et al. Exploration of transcriptional regulation network between buffalo oocytes and granulosa cells and its impact on different diameter follicles. BMC Genomics. 2024;25:1004. 39462339 10.1186/s12864-024-10912-z PMC11515274

[8] Zhang Y, Yan Z, Qin Q, Nisenblat V, Chang HM, Yu Y, et al. Transcriptome landscape of human folliculogenesis reveals oocyte and granulosa cell interactions. Mol Cell. 2018;72(6):1021–34. 30472193 10.1016/j.molcel.2018.10.029

[9] Monniaux D, Michel P, Postel M, Clément F. Multi-scale modelling of ovarian follicular development: From follicular morphogenesis to selection for ovulation. Biol Cell. 2016;108(6):149–60. 26856895 10.1111/boc.201500087

[10] Cinco R, Malott K, Lim J, Ortiz L, Pham C, Del Rosario A, et al. Decreased glutathione synthesis in granulosa cells, but not oocytes, of growing follicles decreases fertility in mice. Biol Reprod. 2024;111(5):1097–106. 10.1093/biolre/ioae124 PMC11565241 39151022

[11] Hoque SAM, Kawai T, Zhu Z, Shimada M. Mitochondrial protein turnover is critical for granulosa cell proliferation and differentiation in antral follicles. J Endocr Soc. 2018;3(2):324–39. 30652133 10.1210/js.2018-00329 PMC6330174

[12] Hoque SAM, Umehara T, Kawai T, Shimada M. Adverse effect of superoxide-induced mitochondrial damage in granulosa cells on follicular development in mouse ovaries. Free Radic Biol Med. 2021;163:344–55. 33385538 10.1016/j.freeradbiomed.2020.12.434

[13] Hou Y, Hu J, Li J, Li H, Lu Y, Liu X. MFN2 regulates progesterone biosynthesis and proliferation of granulosa cells during follicle selection in hens. J Cell Physiol. 2024;239(1):51–66. 37921053 10.1002/jcp.31143

[14] Yan MQ, Zhu BH, Liu XH, Yang YM, Duan XY, Wang Y, et al. Mitoguardin 1 and 2 promote granulosa cell proliferation by activating AKT and regulating the Hippo-YAP1 signaling pathway. Cell Death Dis. 2023;14(11):779. 38012141 10.1038/s41419-023-06312-y PMC10682431

[15] Shen H, Qi X, Hu Y, Wang Y, Zhang J, Liu Z, et al. Targeting sirtuins for cancer therapy: epigenetics modifications and beyond. Theranostics. 2024;14(17):6726–67. 39479446 10.7150/thno.100667 PMC11519805

[16] Peng X, Ni H, Kuang B, Wang Z, Hou S, Gu S, et al. Sirtuin 3 in renal diseases and aging: From mechanisms to potential therapies. Pharmacol Res. 2024;206:107261. 38917912 10.1016/j.phrs.2024.107261

[17] Zhu J, Yang Q, Li H, Wang Y, Jiang Y, Wang H, et al. Sirt3 deficiency accelerates ovarian senescence without affecting spermatogenesis in aging mice. Free Radic Biol Med. 2022;193(Pt 2):511–25. 36336229 10.1016/j.freeradbiomed.2022.10.324

[18] Ge Y, Wu X, Cai Y, Hu Q, Wang J, Zhang S, et al. FNDC5 prevents oxidative stress and neuronal apoptosis after traumatic brain injury through SIRT3-dependent regulation of mitochondrial quality control. Cell Death Dis. 2024;15(5):364. 38802337 10.1038/s41419-024-06748-w PMC11130144

[19] Hu Y, Zheng Y, Liu C, You Y, Wu Y, Wang P, et al. Mitochondrial MOF regulates energy metabolism in heart failure via ATP5B hyperacetylation. Cell Rep. 2024;43(10):114839. 39392752 10.1016/j.celrep.2024.114839

[20] Zhang A, Pan Y, Wang H, Ding R, Zou T, Guo D, et al. Excessive processing and acetylation of OPA1 aggravate age-related hearing loss via the dysregulation of mitochondrial dynamics. Aging Cell. 2024;23(4):e14091. 38267829 10.1111/acel.14091 PMC11019136

[21] Zhao S, Gong J, Wang Y, Heng N, Wang H, Hu Z, et al. Sirtuin 3 regulation: a target to alleviate β-hydroxybutyric acid-induced mitochondrial dysfunction in bovine granulosa cells. J Anim Sci Biotechnol. 2023;14:18. 36788581 10.1186/s40104-022-00825-w PMC9926763

[22] Du R, Gao Y, Yan C, Ren X, Qi S, Liu G, et al. Sirtuin 1/sirtuin 3 are robust lysine delactylases and sirtuin 1-mediated delactylation regulates glycolysis. iScience. 2024;27(10):110911. 39351192 10.1016/j.isci.2024.110911 PMC11440250

[23] Wang L, Wang Y, Li B, Zhang Y, Song S, Ding W, et al. BMP6 regulates AMH expression via SMAD1/5/8 in goat ovarian granulosa cells. Theriogenology. 2023;197:167–76. 36525856 10.1016/j.theriogenology.2022.11.045

[24] Liu J, Guo C, Fu J, Liu D, Liu G, Sun B, et al. Identification and functional analysis of circRNAs during goat follicular development. Int J Mol Sci. 2024;25(14):7548. 39062792 10.3390/ijms25147548 PMC11277404

[25] Li H, Chen J, Liu J, Lai Y, Huang S, Zheng L, et al. CPT2 downregulation triggers stemness and oxaliplatin resistance in colorectal cancer via activating the ROS/Wnt/β-catenin-induced glycolytic metabolism. Exp Cell Res. 2021;409(1):112892. 34688609 10.1016/j.yexcr.2021.112892

[26] Nie R, Zhang W, Tian H, Li J, Ling Y, Zhang B, et al. Regulation of follicular development in chickens: WIF1 modulates granulosa cell proliferation and progesterone synthesis via Wnt/β-catenin signaling pathway. Int J Mol Sci. 2024;25(3):1788. 38339068 10.3390/ijms25031788 PMC10855829

[27] Liu J, Feng G, Guo C, Li Z, Liu D, Liu G, et al. Identification of functional circRNAs regulating ovarian follicle development in goats. BMC Genomics. 2024;25:893. 39342142 10.1186/s12864-024-10834-w PMC11439210

[28] Silpa MV, Naicy T, Aravindakshan TV, Radhika G, Boswell A, Mini M. Sirtuin3 (SIRT3) gene molecular characterization and SNP detection in prolific and low prolific goat breeds. Theriogenology. 2018;122:47–52. 30227304 10.1016/j.theriogenology.2018.09.008

[29] Pacella-Ince L, Zander-Fox DL, Lan M. Mitochondrial SIRT3 and its target glutamate dehydrogenase are altered in follicular cells of women with reduced ovarian reserve or advanced maternal age. Hum Reprod. 2014;29(7):1490–9. 24771001 10.1093/humrep/deu071

[30] Wu S, Gan M, Wang Y, Pan Y, He Y, Feng J, et al. Copper mediated follicular atresia: Implications for granulosa cell death. J Hazard Mater. 2024;477:135391. 39106724 10.1016/j.jhazmat.2024.135391

[31] Wang J, Chu K, Wang Y, Li J, Fu J, Zeng YA, et al. Procr-expressing granulosa cells are highly proliferative and are important for follicle development. iScience. 2021;24(2):102065. 33644709 10.1016/j.isci.2021.102065 PMC7889980

[32] Zheng Y, Ma L, Liu N, Tang X, Guo S, Zhang B, et al. Autophagy and apoptosis of porcine ovarian granulosa cells during follicular development. Animals. 2019;9(12):1111. 31835576 10.3390/ani9121111 PMC6940823

[33] Huang L, Yuan H, Shi S, Song X, Zhang L, Zhou X, et al. CLOCK inhibits the proliferation of porcine ovarian granulosa cells by targeting ASB9. J Anim Sci Biotechnol. 2023;14:82. 37280645 10.1186/s40104-023-00884-7 PMC10245596

[34] Ungurianu A, Zanfirescu A, Margină D. Sirtuins, resveratrol and the intertwining cellular pathways connecting them. Ageing Res Rev. 2023;88:101936. 37116286 10.1016/j.arr.2023.101936

[35] Chen S, Yang X, Yu M, Wang Z, Liu B, Liu M, et al. SIRT3 regulates cancer cell proliferation through deacetylation of PYCR1 in proline metabolism. Neoplasia. 2019;21(7):665–75. 31108370 10.1016/j.neo.2019.04.008 PMC6526305

[36] Liu X, Xie X, Li D, Liu Z, Zhang B, Zang Y, et al. Sirt3-dependent regulation of mitochondrial oxidative stress and apoptosis contributes to the dysfunction of pancreatic islets after severe burns. Free Radic Biol Med. 2023;198:59–67. 36738799 10.1016/j.freeradbiomed.2023.01.027

[37] Matoba H, Fujii C, Maruyama K, Kawakubo M, Momose M, Sano K, et al. Sirt3 regulates proliferation and progesterone production in leydig cells via suppression of reactive oxygen species. Endocrinology. 2024;165(4):bqae017. 38354290 10.1210/endocr/bqae017

[38] Chen Y, Yang Z, Bai J, Wang X, Yuan Q, Mi Y, et al. Bioactive lignan honokiol alleviates ovarian oxidative stress in aging laying chickens by regulating SIRT3/AMPK pathway. Antioxidants. 2024;13(3):377. 38539910 10.3390/antiox13030377 PMC10967992

[39] Yi X, Guo W, Shi Q, Yang Y, Zhang W, Chen X, et al. SIRT3-dependent mitochondrial dynamics remodeling contributes to oxidative stress-induced melanocyte degeneration in vitiligo. Theranostics. 2019;9(6):1614–33. 31037127 10.7150/thno.30398 PMC6485185

[40] Zhang J, Wang H, Slotabec L, Cheng F, Tan Y, Li J. Alterations of SIRT1/SIRT3 subcellular distribution in aging undermine cardiometabolic homeostasis during ischemia and reperfusion. Aging Cell. 2023;22(9):e13930. 37537789 10.1111/acel.13930 PMC10497814

[41] Zhang Q, Ren J, Wang F, Pan M, Cui L, Li M, et al. Mitochondrial and glucose metabolic dysfunctions in granulosa cells induce impaired oocytes of polycystic ovary syndrome through Sirtuin 3. Free Radic Biol Med. 2022;187:1–16. 35594990 10.1016/j.freeradbiomed.2022.05.010

[42] Fan X, Wang Y, Cai X, Shen Y, Xu T, Xu Y, et al. CPT2 K79 acetylation regulates platelet life span. Blood Adv. 2022;6(17):4924–35. 35728063 10.1182/bloodadvances.2021006687 PMC9631617

[43] Wu M, Tan J, Cao Z, Cai Y, Huang Z, Chen Z, et al. Sirt5 improves cardiomyocytes fatty acid metabolism and ameliorates cardiac lipotoxicity in diabetic cardiomyopathy via CPT2 de-succinylation. Redox Biol. 2024;73:103184. 38718533 10.1016/j.redox.2024.103184 PMC11091707

[44] Mao Y, Zhang J, Zhou Q, He X, Zheng Z, Wei Y, et al. Hypoxia induces mitochondrial protein lactylation to limit oxidative phosphorylation. Cell Res. 2024;34(1):13–30. 38163844 10.1038/s41422-023-00864-6 PMC10770133

[45] Choi J, Smith DM, Scafidi S, Riddle RC, Wolfgang MJ. Carnitine palmitoyltransferase 1 facilitates fatty acid oxidation in a non-cell-autonomous manner. Cell Rep. 2024;43(12):115006. 39671290 10.1016/j.celrep.2024.115006 PMC11726389

[46] Li J, Ni Y, Zhang Y, Liu H. GBA3 promotes fatty acid oxidation and alleviates non-alcoholic fatty liver by increasing CPT2 transcription. Aging (Albany NY). 2024;16(5):4591–608. 38428407 10.18632/aging.205616 PMC10968678

[47] Pereyra AS, Rajan A, Ferreira CR, Ellis JM. Loss of muscle carnitine palmitoyltransferase 2 prevents diet-induced obesity and insulin resistance despite long-chain acylcarnitine accumulation. Cell Rep. 2020;33(6):108374. 33176143 10.1016/j.celrep.2020.108374 PMC7680579

[48] Dikalov S, Panov A, Dikalova A. Critical role of mitochondrial fatty acid metabolism in normal cell function and pathological conditions. Int J Mol Sci. 2024;25(12):6498. 38928204 10.3390/ijms25126498 PMC11203650

[49] Liu L, Xie B, Fan M, Candas-Green D, Jiang JX, Wei R, et al. Low-level saturated fatty acid palmitate benefits liver cells by boosting mitochondrial metabolism via CDK1-SIRT3-CPT2 cascade. Dev Cell. 2020;52(2):196–209. 31866205 10.1016/j.devcel.2019.11.012 PMC6996588

[50] Tang M, Dong X, Xiao L, Tan Z, Luo X, Yang L, et al. CPT1A-mediated fatty acid oxidation promotes cell proliferation via nucleoside metabolism in nasopharyngeal carcinoma. Cell Death Dis. 2022;13(4):331. 35411000 10.1038/s41419-022-04730-y PMC9001659

[51] Li D, Zhou L, Liu Z, Zhang Z, Mao W, Shi W, et al. FTO demethylates regulates cell-cycle progression by controlling CCND1 expression in luteinizing goat granulosa cells. Theriogenology. 2024;216:20–9. 38154203 10.1016/j.theriogenology.2023.12.029

[52] Xu X, Pan Y, Zhan L, Sun Y, Chen S, Zhu J, et al. The Wnt/β-catenin pathway is involved in 2,5-hexanedione-induced ovarian granulosa cell cycle arrest. Ecotoxicol Environ Saf. 2023;268:115720. 10.1016/j.ecoenv.2023.115720 37995618

