# Supplementary material for: SIRT3 mediates CPT2 delactylation to enhance mitochondrial function and proliferation in goat granulosa cells
Source: J Anim Sci Biotechnol. 2025 Jul 17;16:101. doi: 10.1186/s40104-025-01231-8 (PMC12269156; doi:10.1186/s40104-025-01231-8)
Supplement: Supplementary file 1 — Additional file 1. Table S1. Primer information used for quantitative real-time PCR. [file 40104_2025_1231_MOESM1_ESM.docx]

**Table S1** Primer information

| **Primer name** | **GenBank accession No.** | **Sequence 5'→3'** | **PCR size, bp** | **Application** |
| --- | --- | --- | --- | --- |
| *PCNA*-Forward | XM_005688167.3 | TGGTCCAGGGCTCCATCTTGAAG | 115 | RT-PCR |
| *PCNA*-Reverse |  | TGAGACGAGTCCATGCTCTGTAGG |  |  |
| *BAX*-Forward | XM_018062750.1 | GGCTGGACATTGGACTTCCTTCG | 148 | RT-PCR |
| *BAX*-Reverse |  | TGGTGAGCGAGGCGGTGAG |  |  |
| *BCL2*-Forward | NM_001314213.1 | TGTGGATGACCGAGTACCTGAACC | 148 | RT-PCR |
| *BCL2*-Reverse |  | GCCAGACTGAGCAGTGCCTTC |  |  |
| *Caspase3*-Forward | NM_001286089.1 | GACAGTGGTTCTGAGGACGACATG | 100 | RT-PCR |
| *Caspase3*-Reverse |  | TTCGCCAGGAAAAGTAACCAGGTG |  |  |
| *SIRT1*-Forward | NM_001314319.1 | ACCAGTAGCACTAATTCCAAGTTCCATAC | 149 | RT-PCR |
| *SIRT1*-Reverse |  | CGCCACCTAACCTATGACACAGTTC |  |  |
| *SIRT2*-Forward | XM_018062368.1 | CCCTTGCCGACCTCCTTGG | 92 | RT-PCR |
| *SIRT2*-Reverse |  | GGACCGAGATGCCGACTGG |  |  |
| *SIRT3*-Forward | XM_005700087.3 | CCTGCTGCATCTGGCTGACTTC | 102 | RT-PCR |
| *SIRT3*-Reverse |  | CTCCGCACGGCATCAGACAAG |  |  |
| *SIRT4*-Forward | XM_005691562.3 | GCTCCCGATGGCGATGTCTTTC | 129 | RT-PCR |
| *SIRT4*-Reverse |  | CTTGTCAGGCTTCACCGTGTCC |  |  |
| *SIRT5*-Forward | XM_018039215.1 | CAGGTGGTGGTCATAACTCAGAACATC | 121 | RT-PCR |
| *SIRT5*-Reverse |  | CAGCCACAACTCCACAAGAGGTAC |  |  |
| *SIRT6*-Forward | XM_005682565.3 | TCTTCCTCGTCCCACCCTATTTCC | 85 | RT-PCR |
| *SIRT6*-Reverse |  | CCGACATCCTCACTGCACCAAC |  |  |
| *SIRT7*-Forward | XM_018065442.1 | TCAGCACGGCAGCCTCTATCC | 96 | RT-PCR |
| *SIRT7*-Reverse |  | CACTCAGGTCAGCAGCACTAACAC |  |  |
| *SOD1*-Forward | NM_001285550.1 | CCTGGGCAATGTGAAGGCTGAC | 107 | RT-PCR |
| *SOD1*-Reverse |  | CCACCATCGTGCGGCCAATG |  |  |
| *SOD2*-Forward | XM_018053428.1 | ACGTCGCCGAGGAGAAGTACC | 141 | RT-PCR |
| *SOD2*-Reverse |  | CACCGTTGGGGCTCAGATTTGTC |  |  |
| *CAT*-Forward | XM_005690077.3 | ACACAGGCACATGAACGGATATGG | 150 | RT-PCR |
| *CAT*-Reverse |  | GGGTCTTCGTGGGCAAGTCTTG |  |  |
| *GPX1*-Forward | XM_005695962.3 | GTGCGAGGTCAATGGCGAGAAG | 91 | RT-PCR |
| *GPX1*-Reverse |  | CATGAGAGCAGTGGCGTCGTC |  |  |
| *PGC-1α*-Forward | NM_001285631.1 | CCGACCCGTGCTACCTGAGAG | 130 | RT-PCR |
| *PGC-1α*-Reverse |  | GCTTGACTGGGATGACCGAAGTG |  |  |
| *NRF1*-Forward | XM_018046930.1 | CCTGATGGCACTGTCTCGCTTATC | 88 | RT-PCR |
| *NRF1*-Reverse |  | TGACTGTGGTCGGCAATTCTGAAG |  |  |
| *TFAM*-Forward | XM_005699371.3 | GCTGTGGAGGGAACTTCCTGATTC | 80 | RT-PCR |
| *TFAM*-Reverse |  | CTTCCTTGTATGCCTGCCAGTCTG |  |  |
| *CPT2*-Forward | XM_018044297.1 | GGTATGGTGCCTACTTGGTCAATG | 118 | RT-PCR |
| *CPT2*-Reverse |  | GCCTTGTCATCGGTGAAGAGTTC |  |  |
| *ACSL1*-Forward | XM_005698716.3 | GACTCCTACGGCAGTGATCTCC | 103 | RT-PCR |
| *ACSL1*-Reverse |  | GCTTCTGTCTGTTGGCTCTTCC |  |  |

| **Primer name** | **GenBank accession No.** | **Sequence 5'→3'** | **PCR size, bp** | **Application** |
| --- | --- | --- | --- | --- |
| *CPT1A*-Forward | XM_018043311.1 | CGACCATGTACGCCAAGATTGAC | 112 | RT-PCR |
| *CPT1A*-Reverse |  | ACGCCGCTCACCACCTTC |  |  |
| *ACOX1*-Forward | XM_018063769.1 | GGCTATGATGAGATGGATAATGGCTAC | 92 | RT-PCR |
| *ACOX1*-Reverse |  | GGCTTCACCTGGGCATGTTTC |  |  |
| *ACADS*-Forward | XM_018061122.1 | CGTCGCCTACATACCGTCTACC | 113 | RT-PCR |
| *ACADS*-Reverse |  | ACCTGGGCTGCTATGGGAAAG |  |  |
| *ECHS1*-Forward | XM_018041274.1 | TTGAGACAGTGGTTGAAGAAGCC | 96 | RT-PCR |
| *ECHS1*-Reverse |  | CTGCGTTCACCGACTCTTTGG |  |  |
| *ACTB*-Forward | NM_001314342.1 | TCTGGCACCACACCTTCTACAAC | 112 | RT-PCR |
| *ACTB*-Reverse |  | ATCTGGGTCATCTTCTCACGGTTG |  |  |
