# Supplementary material for: SIRT3 mediates CPT2 delactylation to enhance mitochondrial function and proliferation in goat granulosa cells
Source: J Anim Sci Biotechnol. 2025 Jul 17;16:101. doi: 10.1186/s40104-025-01231-8 (PMC12269156; doi:10.1186/s40104-025-01231-8)
Supplement: Supplementary file 2 — Additional file 2. Table S2. Antibodies used in this study. [file 40104_2025_1231_MOESM2_ESM.docx]

**Table S2** Antibody information

| **Antibody name** | **Dilution ratio** | **Source** | **Cat. #** |
| --- | --- | --- | --- |
| PCNA | 1:5000 | Proteintech | 10205-2-AP |
| BAX | 1:3000 | Proteintech | 50599-2-Ig |
| BCL2 | 1:1000 | Proteintech | 26593-1-AP |
| Caspase3 | 1:1000 | Proteintech | 19677-1-AP |
| SIRT1 | 1:3000 | Proteintech | 13161-1-AP |
| SIRT2 | 1:5000 | Proteintech | 66410-1-Ig |
| SIRT3 | 1:2000 | Proteintech | 10099-1-AP |
| SIRT4 | 1:5000 | Proteintech | 66543-1-Ig |
| SIRT5 | 1:3000 | Proteintech | 15122-1-AP |
| SIRT6 | 1:1000 | Proteintech | 13572-1-AP |
| SIRT7 | 1:2000 | Proteintech | 12994-1-AP |
| TFAM | 1:5000 | Proteintech | 22586-1-AP |
| OPA1 | 1:2000 | Proteintech | 27733-1-AP |
| MFN1 | 1:4000 | Proteintech | 13798-1-AP |
| MFN2 | 1:5000 | Proteintech | 12186-1-AP |
| DRP1 | 1:5000 | Proteintech | 12957-1-AP |
| FIS1 | 1:4000 | Proteintech | 10956-1-AP |
| CPT2 | 1:4000 | Proteintech | 26555-1-AP |
| K-Lac | 1:1000 | PTM Bio | PTM-1401RM |
| CCND1 | 1:1000 | Beyotime | AF0126 |
| β-catenin | 1:1000 | Servicebio | GB150016 |
| Tubulin | 1:20000 | Proteintech | 66031-1-Ig |
| β-actin | 1:20000 | Proteintech | 66009-1-Ig |
| VDAC1 | 1:3000 | Proteintech | 55259-1-AP |
| LDHA | 1:1000 | Bioworld | BS6179 |
| LDHB | 1:1000 | Bioworld | BS70831 |
| HRP-conjugated Goat Anti-Mouse IgG(H+L) | 1:5000 | Proteintech | SA00001-1 |
| HRP-conjugated Goat Anti-Rabbit IgG(H+L) | 1:5000 | Proteintech | SA00001-2 |
